# Exposure-based Interventions for Chronic Pain and Bodily Symptoms: A Special Interest Meeting Report

**DOI:** 10.1016/j.brat.2026.104998

**Published:** 2026-02-24

**Authors:** Lea Schemer, Eveliina Glogan, Rachel Sjouwerman, Robert Ahm, Yoni K. Ashar, Yannick Boddez, Katja Boersma, J.P. Caneiro, Rilana F.F. Cima, Marlies den Hollander, Ida Flink, Julia A. Glombiewski, Marielle Goossens, Lauren E. Harrison, Maria Hedman-Lagerlöf, Ivan P.J. Huijnen, Skye King, Albère Köke, Steven J. Linton, Brjánn Ljótsson, Ann Meulders, Peter O’Sullivan, Jenny Riecke, Saskia Scholten, Laura E. Simons, Rob J.E.M. Smeets, Kim G. Smolderen, Caroline van Heugten, Charlotte C.M. van Laake-Geelen, Thijs van Meulenbroek, Jeanine A. Verbunt, Rikard K. Wicksell, Hedvig Zetterberg, Johan W.S. Vlaeyen

**Affiliations:** a Department of Clinical Psychology and Psychotherapy, RPTU University Kaiserslautern-Landau, Landau, Germany; b Health Psychology, Faculty of Psychology and Educational Sciences, KU Leuven, Leuven, Belgium; c Department of Psychology, University of Southern Denmark, Odense, Denmark; d Department of Medicine, University of Colorado Anschutz Medical Campus, Aurora, CO, USA; e Department of Experimental-Clinical and Health Psychology, Ghent University, Ghent, Belgium; f Center for Health and Medical Psychology, School of Behavioral, Social and Legal Sciences, Örebro University, Örebro, Sweden; g Body Logic Physiotherapy, Perth, Western Australia, Australia; h School of Allied Health, Curtin University, Perth, Western Australia, Australia; i Department of Rehabilitation Medicine, Research School CAPHRI, Maastricht University, Maastricht, the Netherlands; j Centre of Expertise in Rehabilitation and Audiology, Adelante Groep, Hoensbroek, the Netherlands; k Department of Social and Psychological Studies, Karlstad University, Karlstad, Sweden; l Centre for Psychiatry Research, Stockholm Health Care Services, Region Stockholm, Sweden; m Department of Anesthesiology, Perioperative, and Pain Medicine, Stanford University School of Medicine, Stanford, CA, USA; n Department of Clinical Neuroscience, Center for Psychiatry Research, Karolinska Institutet, & Stockholm Health Care Services, Region Stockholm, Stockholm, Sweden; o Research Center Appropriate Rehabilitation Care, Zuyd University of Applied Sciences, Heerlen, the Netherlands; p Department of Neuropsychology and Psychopharmacology, Maastricht University, Maastricht, the Netherlands; q Limburg Brain Injury Center, Maastricht, the Netherlands; r Department of Clinical Neuroscience, Division of Psychology, Karolinska Institutet, Stockholm, Sweden; s Experimental Health Psychology, Department of Clinical Psychological Science, Maastricht University, Maastricht, the Netherlands; t CIR Clinics in Revalidatie, Eindhoven, the Netherlands; u Pain in Motion International Research Group, Brussels, Belgium; v Department of Internal Medicine, Section of Cardiovascular Medicine, Vascular Medicine Outcomes (VAMOS) Lab, Yale School of Medicine, New Haven, CT, USA; w Department of Psychiatry, Psychology Section, Yale School of Medicine, New Haven, CT, USA; x Department of Clinical Neuroscience, Karolinska Institutet, Stockholm, Sweden; y Pain Clinic, Capio S:t Goran’s Hospital, Stockholm, Sweden; z Department of Psychology and Social Work, Mid Sweden University, Östersund, Sweden

**Keywords:** Exposure-based treatments, Chronic pain, Bodily symptoms, Interdisciplinary care, Consolidated framework for implementation research

## Abstract

More than two decades have passed since exposure-based interventions were first applied to four individuals with chronic low back pain reporting pain-related fear. To reflect on the progress made since then, an international Special Interest Meeting gathered experts for two days of active dialogue and discussion focusing on the theoretical foundations of exposure-based interventions and their broader application to bodily symptoms from an interdisciplinary perspective. In a subsequent joint paper, the participants summarized how exposure-based interventions have been applied across clinical settings (psychology, behavioral and rehabilitation medicine), treatment providers (psychologists, physiotherapists, physiatrists), delivery formats (digital and in-person), and treatment adaptations (for different age groups and co-occurring conditions). Beyond chronic pain, emerging applications have also extended to a wider range of bodily symptoms, including chronic neuropathic pain, post-concussion symptoms, tinnitus, female genitopelvic pain, cardiovascular symptoms, and gastrointestinal symptoms. To facilitate implementation in clinical practice, the Consolidated Framework for Implementation Research (CFIR) was used to systematically identify evidence gaps and inform a strategic roadmap for future research. Theoretical models that have shaped the field were examined for their potential to guide future innovation. Continued research is needed to clarify which individuals benefit most within a matched care framework and to identify optimal strategies for implementation in routine practice.

## 1. Introduction

Although exposure-based interventions are a core component of cognitive behavioral therapy (CBT), its application to bodily symptoms developed relatively late. Philips (1987) planted the first seeds for its use arguing that exposure-based interventions could challenge pain-harm expectations, an idea later supported by experimental work showing that individuals tend to overpredict pain but recalibrate expectation following repeated exposures (Rachman & Arntz, 1991). For example, when individuals with low back pain performed four maximal-force exercise bouts, they initially overestimated pain but corrected their expectations based on actual experience (Crombez et al., 1998). Building on this evidence, the first clinical test of graded in vivo exposure-based treatment in chronic pain used a replicated cross-over single-case experimental design (SCED) in four individuals with chronic low back pain and high pain-related fear (Vlaeyen et al., 2001). After a baseline phase, individuals received either exposure-based treatment followed by graded activity or the reverse sequence. Across 63 daily ratings, improvements were observed in fear of movement/(re) injury, fear of pain, and pain catastrophizing only during the exposure phase, independent of sequence.

Following a Special Interest Meeting, this report synthesizes expert knowledge on the progress of exposure-based interventions since their initial application, focusing on their generalization across bodily symptoms, disciplines, and clinical settings. Building on a previous narrative review on the behavioral conceptualization and treatment of chronic pain (Vlaeyen & Crombez, 2020) and a systematic review of interoceptive exposure (Farris et al., 2025), this report expands the scope by adopting an interdisciplinary perspective on exposure-based approaches for chronic pain and other bodily symptoms and broadening exposure targets beyond the traditionally proprioceptive focus to include interoceptive and other stimulus types.

The aims of the Special Interest Meetings were: 1. to integrate expert knowledge on the generalization of exposure-based interventions across bodily symptoms, disciplines, and clinical settings. 2. to develop a research roadmap to support optimization and broader implementation of these interventions.

What are the novelties expressed in this report? More than two decades have passed since exposure-based interventions were first applied to four individuals with chronic low back pain reporting pain-related fear. To reflect on the progress made since then, an international Special Interest Meeting gathered experts for two days of active dialogue and discussion focusing on the theoretical foundations of exposure-based interventions and their broader application to bodily symptoms from an interdisciplinary perspective. In this paper, theoretical models that have shaped the field were examined for their potential to guide future innovation, ensuring that the report is not merely a historical recap but a forward-looking guide. A key novelty of this report is the synthesis of this broad application, consolidating clinical experience and evidence across this diverse range of symptoms in a single, cohesive overview for the first time. The participants summarized how exposure-based interventions have been applied across clinical settings (psychology, behavioral and rehabilitation medicine), treatment providers (psychologists, physiotherapists, physiatrists), delivery formats (digital and in-person), and treatment adaptations (for different age groups and co-occurring conditions). Beyond chronic pain, emerging applications have also extended to a wider range of bodily symptoms, including chronic neuropathic pain, post-concussion symptoms, tinnitus, female genitopelvic pain, cardiovascular symptoms, and gastrointestinal symptoms.

Furthermore, to facilitate implementation in clinical practice, the Consolidated Framework for Implementation Research (CFIR) was used to systematically identify evidence gaps and inform a strategic roadmap for future research. This application of a formal implementation science framework represents another novel contribution, moving beyond a simple list of future directions to structure them within a proven model, thereby providing a clear, actionable pathway for the field.

Although pain and other bodily symptoms can be associated with medical diagnoses, this report takes a behavioral perspective that is agnostic to underlying etiology, focusing instead on patient-reported symptoms across multiple functional systems.

### 1.1. Special Interest Meeting

The two-day meeting, held in Maastricht, the Netherlands, in September 2024, brought together international experts in research and clinical care of exposure-based interventions for chronic pain and other bodily symptoms from Europe, the United States, and Australia. All agreed to be listed as co-authors of this meeting report. Most experts were psychologists (n = 27), with representatives from physiotherapy (n = 5) and rehabilitation medicine (n = 2). Experts invited to the meeting were behavioral researchers or scientist–practitioners authoring relevant publications. While meeting costs were covered, all experts paid their own travel expenses. No honoraria were paid.

The meeting comprised two days of active discussion, complemented by brief, structured presentations on predefined topics, including theoretical underpinnings, lifespan applications, delivery formats, and clinical conditions (see Table S1). The meeting was moderated by LS, EG, RS and JV, and featured two group activities. In the first activity, experts were divided into three groups with the task to develop a conceptualization of exposure-based interventions, then rotated to revise each other’s work. All discussions were transcribed using Otter.ai. In the second activity, participants documented key insights on post-it notes, which were clustered by the moderators into overarching themes and subsequently presented and discussed plenary.

To disseminate key insights, all experts agreed to collaboratively develop a meeting report. Four working groups drafted sections informed by their expertise and literature reviews on theory, exposure-based chronic pain interventions, applications to other bodily symptoms, and challenges and future directions respectively. The group activities informed the formulation of the shared definition and research roadmap. All sections were integrated into a single manuscript and refined through two rounds of feedback. Minor disagreements on whether Acceptance and Commitment Therapy (ACT) can be considered an exposure variant or should be regarded as an independent treatment were resolved through discussion, resulting in the inclusion of ACT as an exposure-based approach. No substantive disagreements arose regarding the shared definition or research roadmap, with experts suggesting only minor language refinements and minor specification requests.

Some limitations should be noted as well. This report synthesizes expert perspectives predominantly from Western contexts, with an overrepresentation of psychologists, and the absence of the perspectives of people with lived experiences of chronic bodily symptoms. This may introduce selection bias, disciplinary bias, as well as cultural bias. The report does not aim to provide a systematic review and meta-analysis, but rather to capture integrative interdisciplinary perspectives, refine conceptual definitions, and generate a research roadmap, rather than exhaustively review published studies. We acknowledge that the literature cited was not identified through a systematic search but reflects the expertise and non-systematic reviews of the co-authors.

### 1.2. Basic assumptions

The behavioral approach to bodily symptoms highlights the interaction of physiological predispositions, learning, and social context in shaping bodily symptoms. Symptoms are viewed not as passive signs of diseases but as modifiable behaviors that can develop and persist through learning. Rather than reflecting a single underlying cause, they often function within networks that mutually reinforce and amplify one another (Linton & Nicholas, 2025; Roefs et al., 2022; Vlaeyen et al., 2022). This perspective supports treatments that focus on modifying factors that contribute to the experience or persistence of bodily symptoms rather than on historical causes, with the aim of disrupting self-perpetuating symptom–behavior cycles.

Originally developed in the context of chronic pain, the Fear-Avoidance Model proposed that maladaptive fear-driven behavioral responses trigger a pathological feedback loop of functional decline, disability, negative affect, and pain amplification (Vlaeyen & Crombez, 2020). *Pain-related fear* is an umbrella term associated with concerns, negative beliefs, increased arousal, and protective behaviors concerning pain and its anticipated consequences. Beliefs may include expectations that pain signals bodily damage, that certain activities or situations will exacerbate pain, that life cannot be resumed with pain, or that pain must strictly be controlled (see Table 1). As a consequence, individuals often avoid external or internal stimuli to prevent these anticipated negative outcomes. Pain-related fear may also be intertwined with other emotions such as irritation, anger, shame, or guilt, which further reinforce avoidance. Similar mechanisms can extend beyond pain to other bodily symptoms.

The construct of pain-related fear and its associated avoidance is supported by laboratory studies (Markfelder & Pauli, 2020), cross-sectional studies in acute and diverse chronic pain populations (Zale et al., 2013), and structural equation modeling that clarifies temporal and causal relations (Gheldof et al., 2010). A systematic review and meta-analysis further demonstrated robust associations between pain-related fear, chronic musculoskeletal pain disorders, and functional impairment, with pain-related fear predicting both chronic pain intensity and progressive disability (Martinez-Calderon et al., 2019).

## 2. Theoretical framework

Exposure-based interventions have a long pedigree in the treatment of anxiety disorders, rooted in the early experimental work of Jones (1924b, 1924a) and, subsequently, Wolpe (1958) on systematic desensitization. Utilizing relaxation as a reciprocal inhibitor of anxiety, individuals were exposed to an incremental series of anxiety-provoking stimuli. As the relaxation response was intended to compete with the anxiety response, a graded format was chosen to keep anxiety levels as low as possible. Interestingly, later studies revealed that comparable effects were obtained without relaxation, suggesting that the exposure to anxiety-inducing stimuli was the essential component of the treatment (Borkovec & Sides, 1979).

From the perspective of learning theory, exposure is often considered to be the clinical analogue of Pavlovian fear extinction: repeated presentations of the conditioned stimulus (CS) in the absence of the aversive unconditioned stimulus (US) leads to a reduction of conditioned responses (CR), such as fear. For example, a person learns that performing a particular movement (CS) does not result in increased pain or bodily harm (no US). Over the last four decades, multiple theoretical accounts have been advanced to explain how exposure-based interventions produce symptom reduction. Here, we provide a brief history by summarizing the main theories that have shaped exposure-based interventions for pain and other bodily symptoms, without aiming to be exhaustive. Building on these theories, an overarching definition of exposure-based interventions for pain and bodily symptoms was developed (see Table 2). Exposure-based interventions differ from other pain treatment approaches such as graded activity or pacing. Whereas graded activity is an operant treatment that targets physical activity levels using quotas, pacing is a behavioral approach aimed at regulating activity to avoid extremes and to disrupt the overactivity–pain–rest cycle.

### 2.1. Emotional Processing Theory

Emotional Processing Theory (Foa & Kozak, 1986; Lang, 1971) conceptualizes fear as a memory structure containing information about feared stimuli, responses, and their meanings. According to this theory, extinction occurs when individuals confront fear-inducing situations and encounter evidence that contradicts their fearful beliefs, allowing new adaptive responses and meanings to develop. As one of the most well-established exposure-based interventions rooted in Emotional Processing Theory, prolonged exposure has demonstrated effectiveness in individuals with severe posttraumatic stress disorder (PTSD; Alpert et al., 2023). Whereas PTSD and anxiety-related disorders typically involve exposure to feared memories or situations, individuals with pain and bodily symptoms are typically exposed to proprioceptive stimuli, such as lifting heavy objects. Emotional Processing Theory posits that treatment success depends on fear activation followed by habituation within and between sessions. However, recent research suggests that these proposed mechanisms do not reliably predict treatment outcomes (Craske et al., 2008; Peterman et al., 2019). Also, research consistently shows that extinguished fears can return under various conditions, contradicting the “corrective learning” assumption of emotional processing theory.

### 2.2. Inhibitory Learning/Retrieval Theory

Inhibitory Learning Theory, later renamed Inhibitory Retrieval Theory (Bouton, 2004; Craske et al., 2014, 2022) represents a major advance in understanding treatment mechanisms by proposing that experiencing CS-no-US pairings creates new inhibitory associations that compete with, rather than erase, existing CS-US associations. This framework accounts for phenomena such as spontaneous recovery (fear returning due to a passage of time after extinction) and renewal (fear returning due to a shift in context, away from the extinction context). A central principle of inhibitory learning theory is maximizing expectation violation, whereby larger mismatches between what is expected and the actual outcomes strengthen new learning. Thus, unlike Emotional Processing Theory, behavioral experiments are a key technique in which a person has their explicit expectations of harm (such as ‘If I lift heavy objects, I will end up with lumbago’) assessed beforehand and then compared to actual outcome during a planned behavior experiment, which is not designed to achieve habituation but rather test the concrete expectations (see Table 1). Given the context-dependence of inhibitory learning, feared movements or events are varied and practiced across multiple settings. Thus, Inhibitory Retrieval Theory complements Inhibitory Learning Theory by emphasizing strategies to enhance retrieval of extinction memories.

### 2.3. Relational Frame Theory

Relational Frame Theory is an operant model of language and cognition that explains how humans learn to relate stimuli and derive new associations beyond direct experience, offering a distinct perspective that can be applied on exposure-based interventions by emphasizing verbal and cognitive processes (Barnes-Holmes et al., 2002). Using operant principles applied to verbal behavior, Relational Frame Theory extends traditional conditioning models by explaining how negative experiences can arise not only from direct experience but also through derived relational networks. For example, a person may fear lifting boxes if told it is harmful, without ever experiencing injury (Bennett et al., 2015). In the context of exposure-based interventions, Relational Frame Theory suggests that behavioral experiments provide new learning experience that creates additional stimulus relations. These new relations become integrated into existing networks and can modify the function of previously feared stimuli.

From a Relational Frame Theory perspective, behavioral experiments can thus be viewed as an operant process aimed at generating new relational frames that alter how stimuli influence emotional and behavioral responses. When successful, this process leads to a transformation of stimulus functions. Beyond behavioral experiments targeting proprioceptive stimuli, the Relational Frame Theory broadens the range of exposure targets (see Table 1). For example, during behavioral experiments targeting interoceptive stimuli, pain sensations can be recontextualized as brain-generated process within broader relational networks, altering the threat function and reducing avoidance. Similarly, behavioral experiments targeting imaginal stimuli that comprise visualizing pain-eliciting movements might work because thoughts and images share relational links with real experiences, allowing changes in derived networks to generalize beyond the imagined context. Relational Frame Theory also accounts for treatment failures when fear-related verbal relations persist despite contradictory experience (De Houwer & Boddez, 2023).

### 2.4. Goal-directed theories

Other theoretical frameworks in understanding behavior during exposure-based interventions are goal-directed theories (Boddez et al., 2020; Moors, 2022; Moors & Fischer, 2019; Scheveneels et al., 2019). These approaches posit that individuals hold beliefs about certain events or stimuli, which may conflict with valued outcomes. Individuals then select behaviors they believe will reduce this conflict. These behaviors may, however, compromise other values that are not considered at the time of behavioral selection. For example, someone who believes that lifting weight could harm their body may choose to have their children walk rather than carry them, thereby preserving their goal of physical integrity at the expense of their goal to connect. This analysis opens the door to additional intervention strategies: Similar to Inhibitory Learning/Retrieval Theory, one strategy is to create prediction errors, such that the situation is no longer perceived as harmful (e.g. lifting my child does not cause back injury). Additional strategies include devaluing the involved goals (e.g., in the case of physical integrity seeing the body as a vessel to be used), questioning the effectiveness of avoidance (e.g., recognizing that not lifting can weaken muscles over time and thus harm physical integrity), highlighting hidden costs to valued outcomes (e.g., reduced opportunities for connecting with a child), and promoting alternative responses (e.g., moving more naturally or fluidly to support both physical safety and other important values including connection with others). In clinical practice, this implies that the targets of behavioral experiments should be selected not only based on the potential for expectation violation but also based on their relevance to goal conflicts.

## 3. Clinical applications

### 3.1. Exposure-based interventions in chronic primary pain

Chronic primary pain is characterized by persistent pain lasting more than three months, accompanied by significant emotional distress and/or functional disability, and affects approximately one in five people in Europe (Rometsch et al., 2025). Clinical applications of exposure-based interventions for chronic primary pain have primarily applied to musculoskeletal conditions, which are the most common chronic pain disorders.

#### 3.1.1. Treatment strategies

Patients with elevated pain-related fear are typically assessed using structured interview, and self-report questionnaires, although one therapist-reported and one performance-based measure also exist (Traxler et al., 2026). Therapy begins with an educational session including a reflection on the paradoxical effects of safety seeking and avoidance, for example by applying the fear-avoidance model (Vlaeyen & Crombez, 2020) to the individual’s situation. Feared daily activities that may serve as proprioceptive targets can be identified using a catalog of everyday movements (Leeuw et al., 2007; Trost et al., 2009). Patients then confront these situations, explicitly verbalizing and testing their negative expectations through behavioral experiments. Safeguards include medical clearance, informed consent, interdisciplinary collaboration (especially in complex cases), gradual progression, and alignment with daily activities and personal goals. Contraindications include red flags such as fractures, infections, cancer, or acute symptoms that require urgent medical attention.

#### 3.1.2. Evidence

Since the first study in 2001, several other SCED studies (Boersma et al., 2004; De Jong et al., 2005; Linton et al., 2002; Schemer et al., 2018; Vlaeyen et al., 2002) and group-based randomized controlled trials (RCTs; Leeuw et al., 2008; Linton et al., 2008; Woods & Asmundson, 2008) have examined the effectiveness of exposure-based interventions, mainly in adults with chronic low back pain. For example, exposure-based treatments have been shown to improve functioning and psychological flexibility more than traditional coping-focused CBT, while CBT better enhanced coping strategies, with no differences in pain intensity (Glombiewski et al., 2018). No significant differences emerged between brief (5 sessions) versus extended (10 sessions) exposure-based treatment, suggesting that characteristics beyond treatment duration may be more critical. No serious adverse effects occurred, and a few adverse events reported were similar to CBT. Interestingly, the benefits from behavioral experiments also generalized to untargeted movements (Riecke et al., 2020).

For youth living with pain, exposure-based interventions targeting mainly proprioceptive stimuli have consistently been shown to reduce disability and other pain-related outcomes (Dekker et al., 2020; Simons et al., 2020), demonstrating effectiveness comparable to interdisciplinary coping-based CBT and physical therapy (Simons et al., 2024). Similarly, exposure-based treatments involving imaginal and interoceptive stimuli reduced pain intensity, school avoidance, and negative pain-related emotions (Gruszka et al., 2018; Hechler et al., 2010).

Exposure-based interventions have also been shown to reduce disability in adults with chronic post-traumatic neck pain (de Jong et al., 2008), work-related upper extremity pain (de Jong et al., 2012), complex regional pain syndrome type 1 (de Jong et al., 2005; den Hollander et al., 2016), and fibromyalgia (Hedman-Lagerlöf et al., 2018; Hedman-Lagerlöf, Gasslander, et al., 2024). In addition, they have demonstrated favorable cost-effectiveness by reducing disability, increasing quality-adjusted life years, and lowering total costs in back pain, complex regional pain syndrome, and fibromyalgia (den Hollander et al., 2018, S. 20; Goossens et al., 2015; Hedman-Lagerlöf et al., 2019, 2025).

### 3.2. Variations in exposure-based interventions

Exposure-based interventions have been remarkably diversified in approach and delivery methods (see Table 3 for an evidence source table; details in Table S2). Cognitive Functional Therapy integrates behavioral experiments targeting proprioceptive stimuli with cognitive restructuring and lifestyle modification. Pain Reprocessing Therapy focuses on retraining pain perception through graded behavioral experiments for proprioceptive (i.e., movements) and interoceptive (i.e., pain) stimuli. Hybrid exposure-based treatments combine traditional exposure techniques with complementary therapeutic elements. Acceptance and Commitment Therapy uses behavioral experiments to promote psychological flexibility by fostering acceptance of internal experiences and promoting value-driven action. The field has further expanded through technological innovations, notably digital exposure-based interventions and virtual reality. This diversity highlights both the adaptability of exposure-based principles and the ongoing refinement of treatments to optimize outcomes for individuals with persistent or recurrent pain. We briefly highlight the treatment strategies and available evidence for each of these variations discussed at the expert meeting.

#### 3.2.1. Cognitive Functional Therapy (CFT)

CFT is an exposure-based approach that addresses the cognitive, emotional, and behavioral contributors to pain disability, combining CBT and exposure techniques within a physical therapy context (O’Sullivan et al., 2018).

##### 3.2.1.1. Treatment strategies.

Rooted in psychology-informed physical therapy, CFT aims to empower individuals to self-manage their condition through three key components: re-conceptualizing pain from a biopsychosocial perspective (*making sense of pain*), increasing awareness of cognitive, emotional, and physical responses and developing pain control strategies via guided behavioral experiments targeting painful, feared or avoided movements and activities (*exposure with control*), and adopting healthy daily habits, including physical activity, sleep, stress management, and social engagement (*lifestyle coaching)* (O’Sullivan et al., 2018).

##### 3.2.1.2. Evidence.

CFT was found effective in several RCTs (Castro et al., 2022; O’Keeffe et al., 2020; Vibe Fersum et al., 2013). In a recent large-scale RCT, CFT with and without movement sensor biofeedback, produced sustained improvements in disability, pain intensity, and related outcomes at 3-, 6-, 12-month, and 3-year follow-ups, compared to usual care (Hancock et al., 2025; Kent et al., 2023). The treatment had large, durable, and clinically important effects on common mechanisms of change, including self-efficacy, pain-related fear, and catastrophizing (Schütze et al., 2025). Observational data showed that CFT also increased forward bending speed, which in turn was linked to reduced pain and disability (Chang et al., 2024), and improved pain catastrophizing and self-efficacy (Chang et al., 2025). It has also yielded cost savings, mostly due to increased work participation, benefited individuals across disability levels, and was highly accepted and safe, with 84% satisfaction and low dropout rates associated with CFT (Hancock et al., 2024; Kent et al., 2023). A recent SCED study also investigated the benefits of CFT in knee osteoarthritis (Caneiro et al., 2024).

#### 3.2.2. Pain Reprocessing Therapy (PRT)

PRT is an exposure-based intervention that integrates cognitive, somatic, and emotion-focused elements. Unlike other exposure-based approaches described here, that primarily focus on reducing pain-related disability or interference, pain reprocessing therapy aims for full recovery, defined as the resolution of pain reports (Lumley & Schubiner, 2019).

##### 3.2.2.1. Treatment strategies.

A central aspect of PRT is helping individuals reframe pain as a non-dangerous, reversible mind-brain process rather than an indicator of tissue damage, thereby reducing pain-related fear and avoidance and weakening pain-threat associations. Treatment includes behavioral experiments targeting pain sensations as interoceptive stimuli while reappraising them as “non-dangerous”, feared postures or movements as proprioceptive stimuli, and techniques to identify and address other affective processes that contribute to pain (e.g., stress, anxiety, and difficult emotions) (Ashar et al., 2022; Tankha et al., 2023).

##### 3.2.2.2. Evidence.

In an RCT comparing PRT, placebo, and care as usual, 66% of individuals randomized to PRT were pain-free or nearly so at post-treatment, versus fewer than 20% in the control groups, with benefits largely maintained through 1-year follow-up (Ashar et al., 2022) and still evident at 5-years follow-up (Ashar et al., 2025). Pain reductions were mediated by decreased harm beliefs, reduced activity avoidance, and a shift in pain attributions from biomedical to mind-brain causes (Ashar et al., 2023). Pilot trials have demonstrated feasibility and acceptability of PRT in migraine and fibromyalgia (Fishbein et al., 2025; Sturgeon et al., 2025).

#### 3.2.3. Hybrid exposure-based treatment

One hybrid exposure-based treatment was developed as a transdiagnostic treatment approach for individuals with chronic pain and co-occurring emotional problems, such as depression and anxiety (Linton, 2013). Its aim is to target the shared elements of pain and emotional problems.

##### 3.2.3.1. Treatment strategies.

This hybrid exposure-based treatment combines behavioral experiments with Dialectical Behavior Therapy (DBT) emotion-regulation techniques, focusing on developing skills to accurately and effectively identify, understand, soothe, and act upon their emotional responses to aversive stimuli (Koerner, 2012). Five treatment phases have specific techniques: establishing relationships and goals, emotion regulation skills, exposure phase, social relationships, and maintenance skills (Bergbom et al., 2025). The exposure phase targets both pain and emotion-related avoidance behaviors, including feared movements and activities as proprioceptive stimuli, but also emotionally demanding social situations as emotional stimuli. As such, the hybrid exposure-based treatment is flexible and tailored to the individual’s needs.

##### 3.2.3.2. Evidence.

After an initial SCED study (Linton & Fruzzetti, 2014), one RCT showed that the hybrid exposure-based treatment produced immediate reductions in pain catastrophizing and interference at post-treatment, and reductions in depression and interference at 9-month follow-up compared to internet-based CBT (Boersma et al., 2019). These effects were mediated by reduced dysregulation of pain and emotions (Södermark et al., 2020). A SCED study is currently ongoing to examine whether the hybrid exposure-based treatment can be implemented in both primary and secondary care (ClinicalTrials.gov Identifier: NCT05082922).

#### 3.2.4. Acceptance and Commitment Therapy (ACT)

Exposure-based interventions have been increasingly integrated within ACT for chronic pain, addressing pain-related fear and avoidance while promoting valued action despite discomfort. Based on Relational Frame Theory, ACT addresses the relationship individuals have with their pain and associated experiences. The model proposes that suffering arises from experiential avoidance and cognitive fusion with pain-related thoughts rather than pain itself. ACT enhances psychological flexibility through six core processes: acceptance, cognitive defusion, present-moment awareness, self-as-context, values clarification, and committed action (McCracken & Vowles, 2014).

##### 3.2.4.1 . Treatment strategies.

ACT employs experiential exercises with exposure techniques playing a central role in addressing avoidance patterns. Behavioral experiments should be explicitly value-driven, with patients engaging in previously avoided activities connecting to personally meaningful life domains. Patients might gradually resume physical activities, social engagements, or occupational tasks abandoned due to pain-related fear. Cognitive defusion techniques help patients observe catastrophic thoughts without behavioral entanglement, while mindfulness practices cultivate present-moment awareness during behavioral experiments without struggle. This integration transforms behavioral experiments from purely fear-reduction exercises into value-based behavioral activation, promoting functional improvement regardless of pain presence. The approach acknowledges that discomfort during behavioral experiments is acceptable when serving valued ends.

##### 3.2.4.2. Evidence.

ACT-based exposure interventions demonstrate promising outcomes in chronic pain populations (Lai et al., 2023). Studies show improvements in pain-related disability, depression, fear-avoidance beliefs, and psychological flexibility, with maintained effects at follow-up (Wicksell et al., 2008, 2009). Psychological flexibility mediates treatment outcomes, supporting theoretical integration of acceptance processes with behavioral experiments (Kemani et al., 2016). Values-based behavioral experiments may enhance treatment engagement and reduce dropout compared to traditional graded exposure, as activities gain personal significance beyond symptom management. However, comparative research directly contrasting ACT-based exposure treatments with conventional approaches remains limited.

#### 3.2.5. Digital exposure-based interventions

Digital health (also referred to as eHealth, mHealth or Internet-delivered treatment), delivered via digital solutions to support self-management, is gaining strong empirical support and offers a cost-effective way to improve access to pain care for individuals in remote and underserved areas with limited access to in-person care, mobility limitations or transportation barriers (Bartels et al., 2025; Hedman-Lagerlöf et al., 2018; Terpstra et al., 2022). The DAHLIA project exemplifies this approach, providing a nationwide digital behavioral health treatment through a national digital health platform (Al Sharaa et al., 2025; Bartels et al., 2022; Taygar et al., 2025).

##### 3.2.5.1. Treatment strategies.

Digital health interventions for chronic pain, delivered via web platforms, apps, or videoconferencing (Bartels et al., 2025), typically consist of multiple modules covering topics, similar to those in exposure-based interventions (Bartels et al., 2025; Hedman-Lagerlöf et al., 2018; Terpstra et al., 2022). Individuals usually engage in an online self-management program while receiving intermittent therapist support via text, phone, or video call, with exposure observed by a therapist via video call.

##### 3.2.5.2. Evidence.

The empirical support for digital health approaches has increased rapidly during the past decade (Eccleston et al., 2014). Existing research shows that digital exposure-based interventions improve resilience to pain and distress, improve functioning and quality of life (Bartels et al., 2025; Hedman-Lagerlöf et al., 2018). Whereas digital exposure-based interventions have not been directly compared with in-person exposure delivery, internet-based CBT (iCBT) for pain has demonstrated comparable psychological and physical benefits to face-to-face CBT (Terpstra et al., 2022). Importantly, recent studies using an individual-level approach to evaluation illustrates that effects vary across patients and outcome variables (Al Sharaa et al., 2025), which points at the relevance of individual-level evaluations of treatment effects. Recently, digital exposure interventions have been successfully applied to youth with chronic pain (Shear et al., 2022; Zetterqvist et al., 2020), and several exposure-based digital interventions are currently under development (de la Vega et al., 2023; Harrison et al., 2022; Miró et al., 2025).

#### 3.2.6. Virtual reality exposure

Virtual reality (VR), a computer-simulated environment replicating real-world experiences, is increasingly being integrated into therapy, including exposure-based interventions for pain. VR allows behavioral experiments when real-world presentations are impractical or impossible, offering the added advantage of precises control over environmental variables.

##### 3.2.6.1. Treatment strategies.

Exposure-based interventions delivered via VR (VRET) use devices such as headsets or 3D glasses to facilitate exposure to feared stimuli. Key elements of VR include being absorbed in a virtual world (*immersion*), the sense of “being there” (*presence*), experiencing the virtual body as one’s own (*embodiment*), and the ability to influence the virtual world (*interactivity*). These unique qualities enable VR to address various dimensions of the pain experience through distraction, enjoyment, increased movement and social interactions.

##### 3.2.6.2. Evidence.

VR interventions generally have shown promise in reducing chronic pain intensity and improving related outcomes. Meta-analyses and systematic reviews reported meaningful improvements in pain, functioning, and psychological well-being, following VR interventions for chronic pain conditions such as fibromyalgia and chronic low back pain (Baker et al., 2022; Goudman et al., 2022; Hu et al., 2024; Pretat et al., 2025). VRET may, in particular, help to break the cycle of pain-related fear and avoidance of movement, though its effects on disability levels remained less clear (Goudman et al., 2022). Most VRET studies in musculoskeletal pain have focused on adults, and long-term benefits and optimal dosing are still uncertain (Hennessy et al., 2020; Trost & Parsons, 2014). Generalization from virtual to real-world settings requires verification.

### 3.3. Generalizations to other bodily symptoms

Exposure-based interventions have also been developed and evaluated in areas beyond chronic primary pain, such as chronic neuropathic pain, female genitopelvic pain, gastrointestinal symptoms, persistent post-concussion symptoms, tinnitus and cardiovascular symptoms. The conditions listed below are not exhaustive but serve as illustrative examples. In treating individuals with bodily symptoms, a key consideration for exposure-based interventions is distinguishing functional from dysfunctional avoidance behaviors. Functional avoidance is context-specific, medically justified, and poses genuine risk, whereas dysfunctional avoidance stems from excessive pain-related fears and overgeneralization.

#### 3.3.1. Chronic neuropathic pain

Chronic neuropathic pain arises from lesions or diseases of the somatosensory nervous system such as painful diabetic neuropathy, small fiber neuropathy, and spinal cord injury (Jensen et al., 2011; Scholz et al., 2019). In these conditions, pain-related fear has been shown to exacerbate both the pain experience and associated disability (Damci et al., 2022; Geelen et al., 2017; Gruener et al., 2018). Individuals with painful diabetic neuropathy, for example, may experience suboptimal treatment outcomes due to premature discontinuation of prescribed interventions. Adherence to physical exercise programs is particularly limited, with dropout rates reaching 45% due to factors such as pressure ulcer development, overuse injuries, and individual lack of interest (Praet et al., 2008). Exercise-related apprehension and fear of painful movement may further contribute to these high dropout rates, limiting the benefits of structured interventions.

##### 3.3.1.1. Treatment strategies.

For exposure-based interventions in chronic neuropathic pain, additional considerations are needed compared to most chronic primary pain conditions (den Hollander et al., 2022; van Laake-Geelen et al., 2019). One challenge is that not all individuals’ avoidance behavior is dysfunctional (Geelen et al., 2017; Kanera et al., 2019). Functional avoidance behaviors that protect against tissue damage include temperature regulation, protective positioning, medication timing and adaptive footwear. During behavioral experiments, clinicians target disproportionate pain-related fears that can be safely challenged, while helping individuals consider diagnosis-related risks (den Hollander et al., 2016). Achieving an optimal balance between reducing dysfunctional avoidance and preserving functional avoidance requires more comprehensive education than is typically necessary for those with chronic primary pain (den Hollander et al., 2022). Additionally, co-occurring impairments may necessitate supplementary interventions beyond targeting pain-related fear (e.g. bladder training).

##### 3.3.1.2. Evidence.

An eight-week, exposure-based treatment protocol was developed for individuals with painful diabetic neuropathy, incorporating customized screening tools and feedback from three patient-partner focus groups (Kanera et al., 2019; van Laake-Geelen et al., 2019). Its effectiveness was evaluated using SCEDs with a 6-month follow-up, which revealed slight, but non-significant, changes in physical activity and disability (van Laake-Geelen et al., 2021). Due to high drop-out rates, these results should be interpreted with caution.

#### 3.3.2. Female genitopelvic pain

Female genitopelvic pain affects nearly a third of women worldwide (Lamvu et al., 2021), and it encompasses conditions such as vulvodynia (chronic vulvar pain without identifiable pathology), vaginismus (characterized by involuntary muscle contractions that interfere with vaginal penetration), and endometriosis (a condition wherein endometrial tissue grows outside the uterus). Historically, genitopelvic pain was primarily attributed to biological factors, including inflammation, vaginal infections, hormonal triggers, and pelvic floor dysfunction (Bergeron et al., 2020), whereas contemporary perspectives adopt a biopsychosocial approach (Mardon et al., 2022). Recent research emphasizes the central role of pain-related fear and avoidance (Chisari et al., 2021; Dewitte & Meulders, 2023), with psychological treatments increasingly guided by the Fear-Avoidance Model.

##### 3.3.2.1. Treatment strategies.

A key challenge in this population is that reducing pain-related fear and avoidance alone is insufficient; enhancing sexual desire and arousal is also necessary for effective outcomes. Therefore, exposure targeting pain-related fear and dysfunctional avoidance is typically complemented by systematic attention to other components, including cognitive techniques for catastrophic beliefs and interventions addressing interpersonal factors such as effective communication (Engman et al., 2022; Ter Kuile et al., 2013). Uniquely, for ethical reasons and to support desire and lubrication, exposure should not provoke genital pain. In fact, a “pain prohibition” approach is typically part of the treatment. Consequently, behavioral experiments focus on approaching fear-inducing stimuli associated with sexual activities rather than the pain itself.

##### 3.3.2.2. Evidence.

Evidence for exposure-based interventions as a standalone treatment for genitopelvic pain remains limited. One notable study reported strong efficacy for therapist-guided behavioral experiments in women with lifelong vaginismus (Ter Kuile et al., 2013). For vulvodynia, exposure-based interventions have only been studied as part of comprehensive CBT protocols, yielding promising outcomes (Engman et al., 2022; Santangelo et al., 2023). In addition, a multimodal treatment protocol in which behavior experiments are a central component is currently being developed with its effects being evaluated through a series of replicated SCED studies (ClinicalTrials.gov Identifier: NCT06981611).

#### 3.3.3. Gastrointestinal symptoms

Disorders of gut-brain interaction are associated with a combination of factors, including disturbances in motility, visceral hypersensitivity, altered mucosal and immune function, dysbiosis of the gut microbiota, and changes in central nervous system processing (Guadagnoli et al., 2025; Sperber, 2021). Among these, irritable bowel syndrome is the most prevalent, affecting approximately 4–9% (Oka et al., 2020). Irritable bowel syndrome is characterized by recurring abdominal pain and accompanied by altered bowel habits, such as diarrhea and/or constipation (Longstreth, 2005).

##### 3.3.3.1. Treatment strategies.

Exposure-based interventions for irritable bowel syndrome place strong emphasis on response prevention. Safety behaviors, such as repeated toilet visits, resting, or taking unprescribed medications, are systematically targeted. Instead, individuals are encouraged to deliberately expose themselves to symptoms by eating certain foods, engaging in physical activity, and entering stressful situations. Compared to interoceptive exposure, less emphasis is placed on bodily symptoms alone. Rather, exposure is primarily conducted in contexts where symptoms are particularly unwanted, such as at work or in social settings. A self-observation component complements behavioral experiments by fostering greater awareness of gastrointestinal symptoms and the cascade of thoughts, feelings, and behavioral urges they precipitate (Ljótsson et al., 2014).

##### 3.3.3.2. Evidence.

A meta-analysis examining nine psychological treatment approaches for irritable bowel syndrome found that only exposure-based interventions (and hypnotherapy) were likely to outperform attention control approaches in reducing irritable bowel symptoms (Axelsson et al., 2023). Exposure-based interventions have been evaluated by research groups in various countries, including Japan (Kikuchi et al., 2022) and the US (Craske et al., 2011). The most extensively studied protocol was developed in Sweden and tested in four RCTs (Ljótsson, Andersson, et al., 2011; Ljótsson et al., 2010, 2014; Ljótsson, Hedman, et al., 2011) and two clinical implementation studies (Wallén et al., 2022, 2025). Mediation studies have shown that the effect of exposure-based approaches on irritable bowel symptoms is driven by reduction in IBS-related fear and avoidance behavior (Hesser et al., 2018; Ljótsson et al., 2013; Wallén et al., 2025; Wolitzky-Taylor et al., 2012), whereas a moderation study suggested that treatment effects are stronger in individuals with higher baseline avoidance (Hesser et al., 2021). Exposure-based interventions have also shown to reduce symptom severity of gut-brain interaction disorder in pediatric samples (Bonnert et al., 2017; Lalouni et al., 2019), with evidence suggesting that reductions in avoidance, symptom-related fear, and parental catastrophizing (Bonnert et al., 2018; Lalouni et al., 2021, 2022) mediated improvements in gut symptoms.

#### 3.3.4. Persistent post-concussion symptoms

Every year, millions of people sustain head injuries, most of which are diagnosed as mild traumatic brain injury or concussion (Cassidy et al., 2014). Concussions can directly lead to symptoms, such as headaches, fatigue and difficulty concentrating, which typically resolve within days to weeks without specific treatment. However, one in three individuals do not fully recover, with symptoms persisting for months or even years (van der Naalt et al., 2017). The Fear-Avoidance Model offers a framework for understanding how post-concussion symptoms develop, are maintained, and sometimes worsen over time (Hecker et al., 2025; Wijenberg et al., 2020). Catastrophic misinterpretation of symptoms can trigger symptom-related fears and concerns disproportionate to the known brain damage, leading to various dysfunctional avoidance behaviors.

##### 3.3.4.1. Treatment strategies.

Based on the Fear-Avoidance Model, a novel treatment protocol was developed for individuals with persistent post-concussion symptoms, consisting of twelve 90-min sessions delivered over five consecutive days (King et al., 2024). The behavior experiments are designed to test negative expectations related to activities that individuals avoided or found bothersome. A typical example is exposure to bright sunlight, which is avoided by continuously wearing sunglasses, outside as well as indoors, or mental activity such as reading complex text in a noisy context. Additional activities outside the sessions, such as fitness or walking, are encouraged. Generalization of avoidance extinction is facilitated through planning home exercises and involving significant others in the program.

##### 3.3.4.2. Evidence.

In an initial series of two SCED studies with four participants each, the exposure-based treatment as described above effectively reduced symptoms and avoidance behaviors while improving satisfaction with daily functioning (King et al., 2024, 2025). Furthermore, a subsequent SCED study testing the effectiveness of a 14-week treatment protocol replicated previous beneficial outcomes (Hecker et al., 2025).

#### 3.3.5. Tinnitus

Tinnitus, or ringing in the ears, is a subjective perceptual experience for which no disease, injury, or pathology in the brain or elsewhere can be detected. Currently, there is no medical or pharmacological cure for tinnitus (Elgoyhen & Langguth, 2010). While tinnitus is relatively common, affecting 16 to 21% of healthy adults (Krog et al., 2010), it becomes a chronic, distressing, and disabling condition to a smaller subgroup (3–6%) (Cima et al., 2011; Davis & Refaie, 2000). The question of why this group enters a vicious circle of incapacitation, while most remain largely unaffected, is likely explained by symptom-related fear and safety behaviors (Cima et al., 2014).

##### 3.3.5.1. Treatment strategies.

To date, exposure-based interventions for tinnitus have been incorporated as part of a comprehensive treatment package alongside techniques such as relaxation, behavioral activation and mindfulness training (Cima et al., 2012). In contrast to sound-based therapies that involve masking, individuals are deliberately encouraged to engage and actively perceive their tinnitus. Behavior experiments involve activities known to exacerbate tinnitus, such as engaging in activities with high contrast: including sound perception (noisy vs silent environments), physical activity (physically active vs physically relaxed engagements), and mental activity (mentally demanding tasks vs. relaxed states).

##### 3.3.5.2. Evidence.

Still limited but gradually accumulating evidence suggests that exposure-based interventions significantly reduce tinnitus distress, alleviate tinnitus-related suffering, improve depressive and anxious mood, and improve quality of life and daily functioning for individuals with chronic bothersome tinnitus (Cima et al., 2012; Fuller et al., 2022; Hesser et al., 2011). Tinnitus-related fear was shown to partly mediate the treatment benefits of exposure-based CBT, as compared to usual care, with respect to quality of life, tinnitus severity and disability (Cima et al., 2018). A Cochrane systematic review concluded that a CBT approach to treat tinnitus is likely effective in reducing the negative impact that tinnitus has on quality of life (Fuller et al., 2022). But specific findings on exposure-based CBT for tinnitus were not reported, because of the scarcity of findings yet.

#### 3.3.6. Cardiovascular symptoms

Approximately 20% of individuals with cardiovascular disease also experience anxiety (Helmark et al., 2021). Across the spectrum of cardiovascular disease, symptoms such as angina, arrhythmias, or heart failure can trigger anxiety that extends beyond cardiac concerns. A key challenge for individuals across conditions is the overlap between cardiac symptoms, anxiety, and normal bodily sensations, such as those occurring during exercise (Ahm et al., 2025; Farris et al., 2019). For example, individuals with ischemic heart disease often become hypervigilant and interpret anxiety or exercise responses as signs of an impending cardiac event (Ahm et al., 2025; Farris et al., 2019).

##### 3.3.6.1. Treatment strategies.

Behavioral experiments combined with psychoeducation about how to distinguish between the symptoms, combined with other CBT techniques have proven effective in the treatment of anxiety in patients with cardiovascular disease (Reavell et al., 2018). Unsupervised patient-directed behavioral experiments may be suboptimal or even contra-indicated in certain cases. Behavioral experiments targeting interoceptive stimuli for instance helps patients discriminate between anxiety-related or exercise-induced sensations and cardiac symptoms. In the context of behavioral experiments targeting physical activity, while monitoring cardiac responses, individuals experience normal cardiovascular sensations, challenge catastrophic beliefs, and learn to differentiate benign exertion and warning signs (Tully et al., 2022). To support adherence and medical supervision, a multidisciplinary approach is recommended (Pedersen et al., 2023).

In cases of trauma or PTSD symptoms after a cardiac event (e.g. myocardial infarction, implantable cardioverter-defibrillator shock), additional trauma-focused exposures can not only improve cardiovascular health but also reduce PTSD symptoms (van den Berk Clark et al., 2022). Adjunctive therapies such as eye movement desensitization reprocessing and imagery rehearsal therapy demonstrate potential for enhancing traumatic memory reprocessing alongside exposure (Arabia et al., 2011; Krakow et al., 1995; Rapelli et al., 2023).

##### 3.3.6.2. Evidence.

Several studies indicate that CBT, including exposure elements, is effective for anxiety in patients with cardiovascular disease (Reavell et al., 2018). In particular, behavioral experiments targeting interoceptive stimuli has been successfully applied in individuals with high anxiety sensitivity in atrial fibrillation (Oser et al., 2021), within a broader exposure framework for atrial fibrillation (Särnholm et al., 2017, 2021), and fear of somatic sensations more generally (Tully et al., 2017 see also Farris et al., 2025, for a review). Emerging evidence also supports the use of eye movement desensitization reprocessing in post-myocardial infarction when patients experience PTSD and anxiety symptoms (Chew et al., 2025).

## 4. Implementation considerations

As described above, a growing body of empirical research suggests that exposure-based interventions are beneficial for chronic pain syndromes, with emerging and preliminary evidence for other bodily symptoms (see Table 3 for an evidence source table; details in Table S2). However, the broader literature indicates that exposure-based interventions often face substantial barriers to implementation (Murray et al., 2022; Pittig et al., 2019; Racz et al., 2024). In pain research, efforts to study the implementation of exposure-based interventions are still limited. Qualitative research suggests that therapists perceive substantial barriers when applying these interventions in routine care (Löfstrand et al., 2024; Simpson et al., 2025), and a recent rehabilitation study in a non-experienced provider team found less than 50% of the patients favorably responded to treatment (Ummels et al., 2025). Even when therapists adhere to core treatment elements and do not use any prohibited elements, delivery quality is often not assessed (Day et al., 2024, 2025). To guide future efforts for the implementation of exposure-based interventions, the team of experts at the international Special Interest Meeting applied the Consolidated Framework for Implementation Research (CFIR) to consider challenges and future directions (Damschroder et al., 2009, 2022). Each domain of CFIR is discussed separately, except for the implementation process, for which we propose activities and strategies within the section of each domain.

### 4.1. Innovation domain

The ‘innovation’ domain comprises exposure-based interventions for chronic pain and bodily symptoms, which we aim to implement. Despite substantial research, several questions remain unanswered.

#### 4.1.1. Who benefits from exposure-based interventions?

Despite growing evidence suggesting the effectiveness of exposure-based interventions beyond pain, a systematic evaluation of their effects across different bodily symptoms is still lacking. Moreover, their application to other bodily symptoms remains largely unexplored. Potential areas for future investigation may include cluster headaches (Fox et al., 2025), cancer-related pain (Black et al., 2025), and post-viral syndromes such as post-COVID syndrome (Milde et al., 2023).

It is also unclear to what extent the presence of pain-related fear is necessary for these interventions to be effective. An important caveat for early research is that most RCTs have applied strict inclusion criteria, enrolling only individuals with elevated scores on pain-related fear questionnaires, based on a mechanistic rationale of the Fear-Avoidance Model. However, it should be noted that recent work has shown that most currently used measures of pain-related fear and avoidance do not meet criteria for recommended use (Traxler et al., 2026), which may limit the validity of such selection strategies. For example, the outcome difference between exposure and graded activity did not depend on the level of pain-related fear (Leeuw et al., 2008). Thus, exposure-based interventions may be effective across broader populations, particularly when delivered in hybrid formats (Boersma et al., 2019; Linton & Fruzzetti, 2014). Similarly, research comparing exposure-based interventions with traditional CBT for fibromyalgia showed that although behavioral avoidance, pain catastrophizing, and hypervigilance varied widely and functioned as key mediators in both treatments, no known variables operated as moderators (Hedman-Lagerlöf, Buhrman, et al., 2024). In the context of irritable bowel syndrome however, analyses suggested that baseline avoidance behavior predicted both treatment effect (high avoidance predicts more symptom improvement) and moderated mediation (high avoidance means more effect is mediated through reduced avoidance), indicating that exposure-based interventions are more suitable when avoidance behavior is pronounced (Hesser et al., 2018, 2021). Thus, it remains unclear which patients benefit from exposure-based interventions and how to select these patients. Evidence is also limited regarding who responds better to different delivery formats, such as in-person versus digital exposures.

**Fig. 1.**
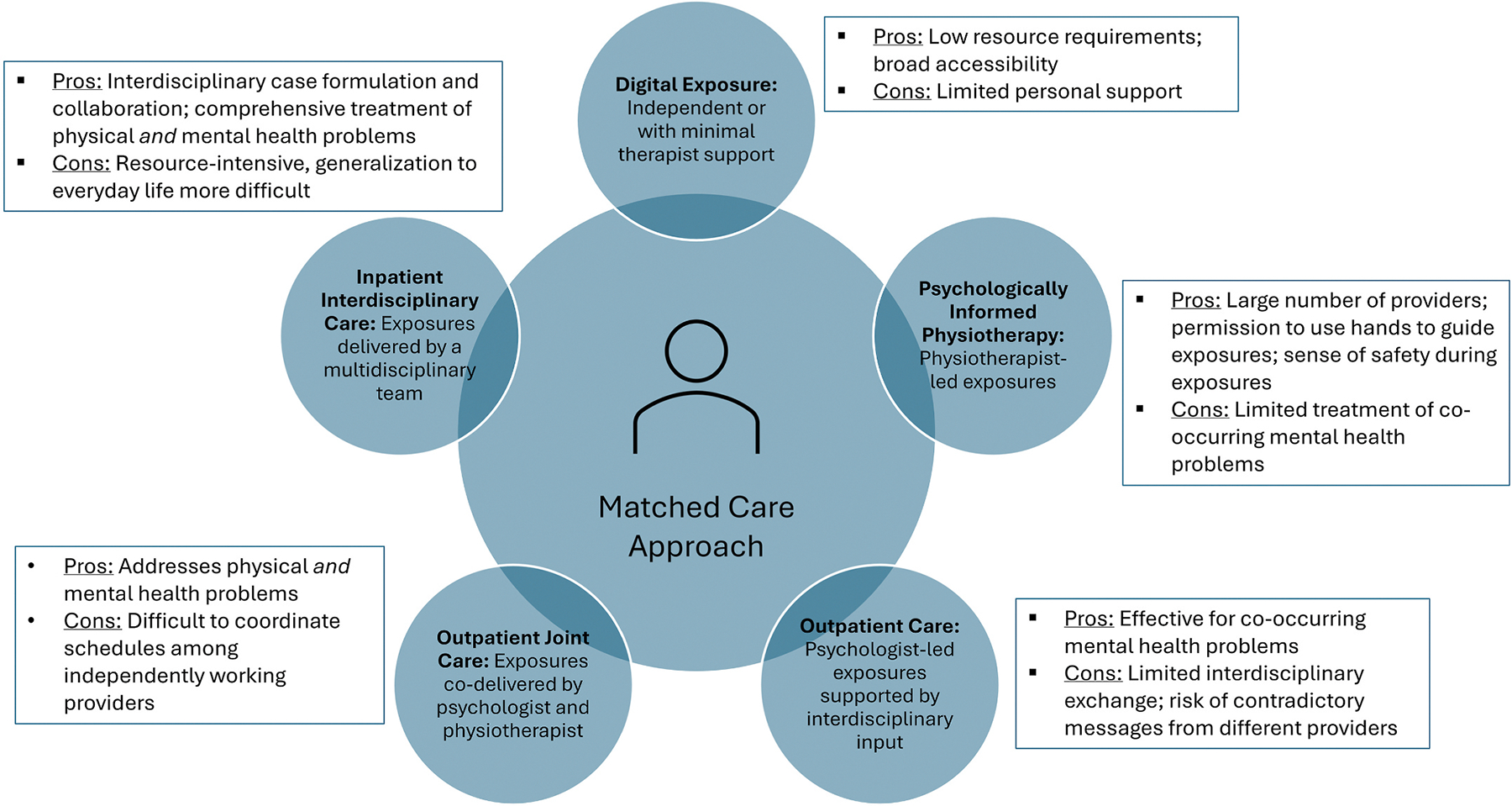
Matched care approach for exposure-based intervention. *Note*. Across research groups, exposure-based interventions for chronic pain and bodily distress have been delivered by different providers in varied settings. Future research could explore a matched-care approach that tailors exposure to the complexity of pain and co-occurring symptoms, using varied intensity, tactics, and interdisciplinary collaboration.

Moving forward, moderated mediation analyses may help to establish evidence-based decision rules, consistent with matched care principles, to guide clinicians in determining which exposure-based intervention is most suitable for whom (see Fig. 1). However, because such average effects may not apply to any given individual (a phenomenon known as non-ergodicity) (Sundström et al., 2025; Vlaeyen & Milde, 2025), an alternative approach is to use SCEDs (Vlaeyen & Morley, 2005). Although several SCED studies have been conducted in this area, they have not been systematically used to address the “what works for whom” question. Such designs may also empower clinician–patient dyads to evaluate treatment progress within routine practice settings (Scholten et al., 2024).

#### 4.1.2. How to maximize outcomes?

Despite encouraging results of outcome studies, exposure-based treatments are often associated with considerable drop-out rates, non-responders and relapse. Over time, the understanding of exposure-based interventions for chronic pain and other bodily symptoms, and the most effective ways to deliver them, has evolved.

Using an Inhibitory Learning/Retrieval Theory perspective, therapeutic strategies to optimize the effects of exposure-based interventions have been proposed and applied to the context of pain (Gatzounis et al., 2021). Preliminary evidence suggests that explicitly verbalizing and testing anticipated negative consequences may enhance response rate and accelerate goal attainment in healthy and subclinical samples (Körfer et al., 2020; Kube et al., 2022; Schemer et al., 2020). Another proposed strategy for challenging anticipated negative outcomes is counterconditioning, in which a CS is paired with a US of opposite valence to change its original meaning and affective valence (Bauer et al., 2024). However, the results of comparing counterconditioning with standard extinction in the context of pain using highly controlled experimental designs have been mixed (Gatzounis et al., 2022; Meulders et al., 2015).

Inspired by goal-directed theories, another yet untested approach is to select targets for behavioral experiments based on both harm expectations and goal conflicts (Schemer et al., 2025). Whereas Inhibitory Learning/Retrieval Theory would prioritize targets with the highest harm expectations to maximize expectation violation, goal-directed theories suggest that targeting behavior with both high harm expectations and high goal conflicts relative to other personal goals may enhance treatment effects.

Recent developments in exposure-based treatment show a shift from primarily targeting proprioceptive stimuli (e.g., lifting) to also addressing other stimuli types (Ashar et al., 2025; Boersma et al., 2019; Gruszka et al., 2018). Relational Frame Theory may help in the future to determine which targets best modify relational networks. For example, interoceptive targets may be the most effective at challenging expectations that pain signals damage and hence needs to be controlled, whereas proprioceptive targets may be the most effective at challenging the expectancy that activity will worsen pain or prevent resuming life (see Table 1).

#### 4.1.3. How to handle safety behavior, precautions, and facilitators?

Concerns about safety often pose a major challenge in implementing exposure-based interventions for chronic pain and bodily symptoms. It is recommended that clinicians discourage unnecessary, mainly fear-driven *safety behaviors* (an approach known as “extinction with response prevention”) because individuals may mistakenly attribute safety to these behaviors, thereby hindering genuine fear extinction (a process known as “protection from extinction” (Lovibond et al., 2000)). However, in some medical conditions, precaution measures may be necessary to reduce real risks and enable individuals to engage in exposure (Sharpe et al., 2022). As discussed for chronic neuropathic pain conditions and cardiovascular diseases, delivering exposure-based interventions in complex cases may benefit from interdisciplinary collaboration to facilitate treatment adherence. For clinicians, interdisciplinary collaboration may help balancing reducing dysfunctional and preserving functional avoidance behaviors, ensuring that exposure remains both effective and feasible.

Beyond medical considerations, evaluating whether a behavior supports or hinders engagement in valued activities could further clarify its function (Sharpe et al., 2022). Building on the motivational dimension, one research group developed an exposure action plan for treating youth with pain that also specifies *exposure facilitators*, such as playing music, to support participation (Simons et al., 2024). Still, more research is needed to clarify the motivational mechanisms underlying these contextual features and their influence on treatment outcomes.

Additional facilitators may include involving significant others in the treatment process. In exposure-based interventions for youth with chronic pain, caregiver involvement is common (Dekker et al., 2020; Simons et al., 2024; Wiertz et al., 2017). In qualitative studies, both youth and caregivers reported gaining practical strategies for managing chronic pain, improved communication and mutual understanding, and, for caregivers, enhanced ability to support their children while learning to let go of control (Corey et al., 2021; Schemer et al., 2023). In contrast, adult treatments typically involve significant others only in special cases, which may overlook the social context of pain and limit opportunities to strengthen outcomes. Future research could explore how systematic involvement of significant others might enhance engagement and support.

### 4.2. Individuals domain

The ‘individuals’ domain concerns the roles and characteristics of those involved in implementing an innovation. Here, we focus on the role of treatment providers. Exposure-based interventions may be prone to therapist drift (Waller, 2009). For mental health problems, a systematic review and meta-analysis showed that health care providers with positive beliefs about exposure-based treatments and prior exposure training were more likely to apply such interventions, whereas older and more anxious therapists were less likely to do so (Langthorne et al., 2023). Another review revealed that negative attitudes about the consequences of exposure-based interventions predicted reduced use (Racz et al., 2024). Notably, general exposure training increased the application of exposure-based interventions in anxiety disorders but not in other conditions. This finding underscores the need for more targeted, practical training for complex disorders such as chronic pain and bodily symptoms. Yet, attitudes toward exposure-based interventions and therapist drift in the context of pain and other bodily symptoms remain largely underexplored.

Although foundational principles for training future pain clinicians have been proposed using exposure-based interventions as an example (Linton et al., 2024), and numerous resources, such as treatment manuals and online trainings are available (e.g., Cognitive Functional Therapy via the Evoolve Pain Care Academy, 2025), evidence of the effectiveness of such trainings is limited. A key limitation of exposure-based intervention training in general is that, while it may reduce clinicians’ negative attitudes toward exposure-based interventions, it often fails to translate into actual changes in treatment behavior (Trivasse et al., 2020). Proposed strategies to address this gap include extended clinician training with supervision (Foa et al., 2020), experiential learning through therapist-conducted exposure tasks (Frank et al., 2020; Kemp et al., 2023), and practical tools such as the Anxiety Coach (Whiteside et al., 2022) and Exposure Guide (Benito et al., 2021). Preliminary evidence supports their effectiveness (Frank et al., 2025). Building on these approaches, future research could investigate which training methods most effectively reduce therapist drift in exposure-based interventions, specifically for chronic pain and bodily symptoms, thereby enhancing treatment outcomes.

### 4.3. Inner setting domain

The ‘inner setting’ refers to the setting in which an innovation is implemented. In some settings, exposure-based interventions have been delivered by an interdisciplinary team with multiple professions collaborating (Linton et al., 2008; Vlaeyen et al., 2001), whereas in others, they are administered solely by psychologists (Glombiewski et al., 2018) or physiotherapists (Kent et al., 2023). Regardless of setting, treatment providers are encouraged to help individuals engage in valued activities while minimizing pain interference and distress through individualized plans (Linton et al., 2024), with each team member supporting this shared goal by addressing avoidance-protection mechanisms at different levels (see Fig. 1). To optimize an interdisciplinary exposure framework, it may also be helpful to consider “de-implementation” strategies, that is the removal of therapeutic practices and treatment components that conflict with the treatment model or common goal to reduce inconsistent messaging across providers (Walsh-Bailey et al., 2021). From a team science perspective, systematically examining the extent to which such collective goal-setting and alignment of treatment models contribute to reduced uncertainty, may help increase adherence, among both treatment recipients and providers (Hess et al., 2023). For teams, continuous intervision probably needs to be available to ensure treatment quality.

### 4.4. Outer setting domain

The outer setting represents the encompassing framework in which the inner setting is embedded. In general, health-care systems have been criticized internationally for over-representing biomedical aspects of pain (Buchbinder et al., 2018). Part of the problem is that treatment recipients and providers were found to be often not aware of other evidence-based treatment options, such as pain psychology (Darnall et al., 2016). Treatment providers also seem hesitant to address the psychological aspects of bodily symptoms in a clinical encounter, despite acknowledging their importance, as suggested by qualitative work (Locher et al., 2023). To address the referral gap in chronic pain, a recent Delphi study developed an ideal referral process and sample phrases for referral conversations, for example, to ensure that upstream referring providers begin to explain pain from a biopsychosocial perspective (Schemer et al., 2024). Interestingly, the Delphi study also revealed potential challenges in the referral process, with experts from different professional backgrounds failing to reach consensus on who should be referred to exposure-based treatments.

Another approach to increase receptiveness may be to optimize the way exposure-based interventions are presented. In PTSD for example, the perception of different names for exposure-based interventions were compared in a representative survey, with simple yet precise treatment names being rated most positively (Larsen et al., 2025). For exposure-based interventions targeting bodily symptoms, various names have already been proposed (e.g. Cognitive Functional Therapy, Pain Reprocessing Therapy) without systematic investigation into how these names are perceived by recipients. In addition, individual patient-partners advocated the use of testimonials, which can provide role models and opportunities to see others facing similar challenges, give insight into the treatment process, and inspire hope (Schemer et al., 2023).

## 5. A roadmap for future research

Future research on exposure-based interventions for chronic pain and bodily symptoms will benefit from a comprehensive research and development road map that addresses several critical domains:

1. Conducting a systematic review and meta-analysis summarizing the current evidence on the (cost)effectiveness of exposure-based treatments in individuals with chronic pain and other bodily symptoms.
2. Identifying the behavioral mechanisms that underly the therapeutic effects of exposure-based interventions, including appropriate methods for measurement.
3. Determining optimal exposure parameters, including not only duration, frequency, and intensity, but also motivational aspects, behavioral targets and relevant social-contexts variables.
4. Developing and validating digital health solutions, including smartphone applications, virtual reality platforms, and wearable devices, for home-based exposure-based treatments, with particular attention to how outcomes generalize when these tools are not available.
5. Expanding research to understudied populations, such as pediatric patients, older adults, individuals with complex comorbidities, people living in low- and middle-income settings, and those from diverse ethnic backgrounds.
6. Investigating exposure-based treatments for bodily symptoms within other medical conditions that remain largely unstudied, such as cluster headaches, cancer-related pain, post-viral syndromes.
7. Establishing evidence-based decision rules, consistent with matched care principles, to guide clinicians in selecting the most appropriate exposure-based intervention for each individual.
8. Increasing person-centered research approaches, such as SCEDs, with a specific focus on the treatment effects and processes for individuals, as group-level findings may not generalize.
9. Examining real-world implementation, including clinician training needs and healthcare system integration, to support the translation of research findings into routine practice and improve access to evidence-based care.

## 6. Conclusion

A key outcome of this international Special Interest Meeting was the recognition that exposure-based interventions appear to generalize across various bodily conditions, health-care settings and providers. If exposure therapy - originally developed for anxiety disorders - helps with diverse bodily symptoms, this suggests that avoidance behaviors and fear-related cognitions may play a larger role in maintaining physical symptoms than traditionally recognized. Exposure may work by helping recalibrate threat detection systems and updating learned associations between certain activities and anticipated harm or symptoms. Exposure-based treatments are often relatively brief, can be delivered by trained therapists rather than requiring expensive medical specialists, and avoid medication costs and side effects. If exposure-based interventions prove effective, they could be offered much earlier in the clinical course, potentially preventing transition from acute to chronic symptoms. Rather than being a last resort after everything else fails, exposure could become part of standard early management.

With the goal to implement exposure-based interventions in routine care, it is of the utmost importance, for both research and clinical purposes, to continue studying their effects, following a systematic, scientifically grounded approach that determines whether, when, in what form, and for whom, exposure-based interventions for bodily symptoms are effective and how clinicians can be best trained to deliver them.

## Supplementary Material

1

## Figures and Tables

**Table 1:** Typical fear-driven cognitive and behavioral responses.

Core expectations	Example thoughts	Avoided stimuli

Pain/bodily symptoms signal body damage.	- ‘Pain during lifting indicates potential back damage’ - ‘Loud noise ruptures my eardrum’ - ‘Wheat and rye products are bad for my colon’ - ‘A fast heartbeat damages my heart’	- Lifting heavy boxes, weights, or children - Exposure to loud noises - Eating wheat- and rye-containing products - Cardiac exercises (e.g., running or cycling)
Certain situations can make pain/bodily symptoms worse.	- ‘If my pain increases, I am injuring myself - ‘If I go to a concert, my tinnitus will worsen’ - ‘If I eat spicy food, I will have diarrhea’ - ‘If I keep doing this, I will get a heart attack’	- Continuing common activities with pain increase - Listening to loud music through headphones - Eating spicy food (e.g., Indian food or salsa sauce) - Engaging in activities beyond the comfort zone
With pain/bodily symptoms, it is impossible to resume life.	- ‘With pain, I cannot do sports’ - ‘With tinnitus, I cannot take part in meaningful activities’ - ‘With IBS, I cannot engage in social activities’ - ‘With chest pain, I cannot enjoy sexual activities’	- Participating in sports (e. g., dancing, tennis, hiking) - Engaging in hobbies (e. g., visiting concerts) - Joining social activities (e.g., restaurants) - Engaging in sexual activities
Pain/bodily symptoms need to be controlled.	- ‘Pain can only be influenced by medical solutions’ - ‘When I settle down, I will notice my tinnitus even more.’ - ‘Without a strict diet, my life will be at risk’ - ‘I will not let chest pain stop me. I will bite the bullet and just tough it out’	- Performing activities without on-demand medication - Avoiding silence or quiet environments - Exploring foods outside rigid dietary rules - Engaging in activities without excessive control or forced endurance

*Note*. Fear-driven cognitive and behavioral responses to pain and bodily symptoms organized around common core expectations. Avoided stimuli often become exposure targets, with negative expectations first verbalized and then tested through behavioral experiments (e.g., “If X happens, Y will follow”).

**Table 2:** Definition of exposure-based interventions for chronic pain and bodily symptoms.

Definition	Description

Core definition	Exposure-based interventions for pain and bodily symptoms involve systematically confronting aversive stimuli associated with pain, bodily symptoms, or distress through learning, with the overarching aim to promote goal-directed action. These interventions specifically target reducing dysfunctional avoidance via behavior experiments.
Component 1: Understanding avoidance	Pain (and likewise other bodily symptoms) typically functions as part of a motivational system that alerts, guides, and energizes behavior to avoid harm. When pain becomes chronic, this system can become dysfunctional, with avoidance intensified by concerns, fears and other emotions such as irritation, anger, shame, or guilt.
Component 2: Promotion of goal-directed action	Exposure-based interventions are goal-directed insofar as they facilitate individuals’ pursuit of personally valued life goals by reducing the discrepancy between current functioning and desired outcomes.
Component 3: Behavior experiments	Grounded in a learning theory framework, behavior experiments are structured as intentional experiences that allow for learning and consolidation of new experiences, by creating expectation violations that challenge pain-harm associations and by encouraging verbal reflection to facilitate generalization. Behavioral experiments may target proprioceptive (e.g., movement-related) but also interoceptive (e.g., pain sensations) and other stimulus types.

*Note*. These definitions emerged from a group exercise at the Special Interest Meeting, where participants were divided into three expert groups. Each group developed their own conceptualization of exposure-based interventions. After 30 min, groups exchanged and revised each others’ work. Discussions were transcribed via Otter.ai. The final definition was synthesized from these contributions and refined during manuscript preparation.

**Table 3:** Evidence source table.

Bodily Symptoms	Uncontrolled Trials	SCED	RCT

**Chronic Primary Pain**	Six uncontrolled studies (half with adults and half with youth) were conducted, with treatment delivered by interdisciplinary providers (n = 2) or psychologists (n = 2); one study was physicianled, and one virtual reality study did not specify the provider.	Twelve SCED studies (one with youth) were conducted, with treatment mainly delivered by interdisciplinary providers (n = 8), followed by psychologists (n = 3) and physiotherapists (n = 1); one study used a digital format.	Seventeen RCTs (three with youth) were conducted, with treatment mainly delivered by interdisciplinary providers (n = 8), followed by psychologists (n = 5) and physiotherapists (n = 4); two studies used a digital format.
**Chronic Neuropathic Pain**	N/A	One SCED study (with adults) was conducted, with treatment delivered by interdisciplinary providers.	N/A
**Female Genitopelvic Pain**	N/A	One SCED study (with adults) was conducted, with treatment delivered by psychologists.	One RCT (with adults) was conducted, with treatment delivered by psychologists.
**Gastrointestinal symptoms**	Two uncontrolled studies (with adults) were conducted, with treatment delivered by psychologists; one study used a digital format.	N/A	Eight RCTs (one with youth) were conducted, with treatment mainly delivered by psychologists (n = 7) and one by interdisciplinary providers; six studies used a digital format.
**Persistent PostConcussion Symptoms**	N/A	Three SCED studies (with adults) were conducted, with treatment delivered by psychologists.	N/A
**Tinnitus**	One uncontrolled study (with adults) was conducted, with treatment delivered by interdisciplinary providers.	N/A	One RCT (with adults) was conducted, with treatment delivered by interdisciplinary providers.
**Cardiovascular Symptoms**	Four uncontrolled studies (with adults) were conducted, with treatment delivered by interdisciplinary providers (n = 2) or psychologists (n = 2).	N/A	N/A

*Note*. This evidence source table categorizes studies by design, also specifying patient age group and treatment provider discipline; digital and virtual reality-based formats are also indicated. Studies were selected by experts based on their expertise and a narrative literature review; no systematic review was conducted. *Uncontrolled trials* lack a control group (e.g., pilot studies, implementation trials). *SCED* = single-case experimental design; *RCT* = randomized controlled trial.

## Data Availability

No data was used for the research described in the article.

## Appendix A. Supplementary data

Supplementary data to this article can be found online at https://doi.org/10.1016/j.brat.2026.104998.
