# Supplementary material for: Exposure-based Interventions for Chronic Pain and Bodily Symptoms: A Special Interest Meeting Report
Source: Behav Res Ther. Author manuscript; Available in PMC 2026 Jun 22. (PMC13284911; doi:10.1016/j.brat.2026.104998)
Supplement: 1 [file NIHMS2178776-supplement-1.docx]

**SUPPLEMENTARY MATERIALS**

| **Table S1.** | Overview of the Expert Input and Group Activities |
| --- | --- |
| **Table S2.** | Studies Included in the Evidence Source Map |

Schemer, L., Glogan, E., Sjouwerman, R., Ahm, R., Ashar, Y. K., Boddez, Y., Boersma, K., Caneiro, J. P., Cima, R. F. F., den Hollander, M., Flink, I., Glombiewski, J. A., Goossens, M., Harrison, L. E., Hedman-Lagerlöf, M., Huijnen, I. P. J., King, S., Köke, A., Linton, S. J., Ljótsson, B., Meulders, A., O’Sullivan, P., Riecke, J., Scholten, S., Simons, L. E., Smeets, R. J. E. M., Smolderen, K. G., van Heugten, C., van Laake-Geelen, C. C. M., van Meulenbroek, T., Verbunt, J. A., Wicksell, R. K., Zetterberg, H., & Vlaeyen, J. W. S. (2026). *Exposure-based interventions for chronic pain and bodily symptoms: A Special Interest Meeting Report* [Supplementary materials]. *Behaviour Research and Therapy*.

**Table S1.**

*Overview of the Expert Input and Group Activities*

| **Theme** | **Topic** | **Expert(s)** |
| --- | --- | --- |
| Expert input | *Theoretical basis* |  |
|  | Fear-Avoidance model, history | Johan Vlaeyen |
|  | History of exposure | Yannick Boddez |
|  | Fear and avoidance | Ann Meulders |
|  | What drives avoidance | Rachel Sjouwerman |
|  | *Exposure across the lifespan* |  |
|  | Exposure in children | Laura Simons |
|  | Exposure in adults | Julia Glombiewski |
|  | *Variations of exposure* |  |
|  | Cognitive functional therapy | JP Caneiro |
|  | Pain reprogramming Therapy | Yoni Ashar |
|  | Digital exposure | Rikard Wicksell |
|  | Hybrid exposure | Katja Boersma |
|  | *Exposure in other health problems* |  |
|  | Irritable bowel syndrome | Brjánn Ljótsson |
|  | Tinnitus | Rilana Cima |
|  | Vulvodynia | Ida Flink |
|  | Cardiovascular problems | Robert Ahm |
|  | Post-concussion syndrome | Caroline Van Heugten |
|  | *Implementation of exposure* |  |
|  | Challenges and opportunities | Marlies Den Hollander |
|  | The doctors view | Charlotte Van Laake  Jeanine Verbunt |
| Group activities | *Panel discussions* |  |
|  | Consensus on a definition | Lea Schemer |
|  | Roadmap for future research | Rachel Sjouwerman Eveliina Glogan  Johan Vlaeyen |

**Table S2.**

*Studies Included in the Evidence Source Map*

| **Citation** | **Study type** | **Condition** | **Categorization** | **Age** | **Provider** | **Setting/Delivery Format** |
| --- | --- | --- | --- | --- | --- | --- |
| Särnholm, Josefin, Helga Skúladóttir, Christian Rück, Susanne S. Pedersen, Frieder Braunschweig, und Brjánn Ljótsson. 2017. „Exposure-Based Therapy for Symptom Preoccupation in Atrial Fibrillation: An Uncontrolled Pilot Study“. *Behavior Therapy* 48(6):808–19. doi:10.1016/j.beth.2017.06.001. | Uncontrolled Trial | Atrial Fibrillation | Cardiovascular Symptoms | Adult | Interdisciplinary | Outpatient |
| Tully, Phillip J., Aline Sardinha, und Antonio E. Nardi. 2017. „A New CBT Model of Panic Attack Treatment in Comorbid Heart Diseases (PATCHD): How to Calm an Anxious Heart and Mind“. *Cognitive and Behavioral Practice* 24(3):329–41. doi:10.1016/j.cbpra.2016.05.008. | Uncontrolled Trial | Panic Attack Treatment in Comorbid Heart Diseases | Cardiovascular Symptoms | Adult | Interdisciplinary | Outpatient |
| Oser, Megan, Amanda Khan, Meghan Kolodziej, Gabriel Gruner, Arthur J. Barsky, und Laurence Epstein. 2021. „Mindfulness and Interoceptive Exposure Therapy for Anxiety Sensitivity in Atrial Fibrillation: A Pilot Study“. *Behavior Modification* 45(3):462–79. doi:10.1177/0145445519877619. | Uncontrolled Trial | Atrial Fibrillation | Cardiovascular Symptoms | Adult | Psychology | Outpatient |
| Särnholm, Josefin, Helga Skúladóttir, Christian Rück, Sofia Klavebäck, Eva Ólafsdóttir, Susanne S. Pedersen, Frieder Braunschweig, und Brjánn Ljótsson. 2021. „Internet-Delivered Exposure-Based Therapy for Symptom Preoccupation in Atrial Fibrillation: Uncontrolled Pilot Trial“. *JMIR Cardio* 5(1):e24524. doi:10.2196/24524. | Uncontrolled Trial | Atrial Fibrillation | Cardiovascular Symptoms | Adult | Psychology | Digital |
| van Laake-Geelen, Charlotte C. M., Rob J. E. M. Smeets, Marielle E. J. B. Goossens, und Jeanine A. Verbunt. 2021. „Effectiveness of exposure in vivo for patients with painful diabetic neuropathy: A pilot study of effects on physical activity and quality of life“. *Journal of Rehabilitation Medicine - Clinical Communications* 4:1–11. doi:10.2340/20030711-1000046. | SCED | Painful Diabetic Neuropathy | Chronic Neuropathic Pain | Adult | Interdisciplinary | Outpatient |
| Wicksell, Rikard K., Josefin Ahlqvist, Annika Bring, Lennart Melin, und Gunnar L. Olsson. 2008. „Can Exposure and Acceptance Strategies Improve Functioning and Life Satisfaction in People with Chronic Pain and Whiplash‐associated Disorders? A Randomized Controlled Trial“. *Cognitive Behaviour Therapy* 37(3):169–82. doi:10.1080/16506070802078970. | RCT | Chronic Pain and Whiplash‐associated Disorders | Chronic Primary Pain | Adult | Interdisciplinary | Outpatient |
| Leeuw, Maaike, Mariëlle E. J. B. Goossens, Gerard J. P. van Breukelen, Jeroen R. de Jong, Peter H. T. G. Heuts, Rob J. E. M. Smeets, Albere J. A. Köke, und Johan W. S. Vlaeyen. 2008. „Exposure in Vivo versus Operant Graded Activity in Chronic Low Back Pain Patients: Results of a Randomized Controlled Trial“. *Pain* 138(1):192–207. doi:10.1016/j.pain.2007.12.009. | RCT | Chronic Back Pain | Chronic Primary Pain | Adult | Interdisciplinary | Outpatient |
| Dekker, Carolien, Mariëlle Goossens, Bjorn Winkens, Silvia Remerie, Caroline Bastiaenen, und Jeanine Verbunt. 2020. „Functional Disability in Adolescents with Chronic Pain: Comparing an Interdisciplinary Exposure Program to Usual Care“. *Children* 7(12):288. doi:10.3390/children7120288. | RCT | Mixed Chronic Pain | Chronic Primary Pain | Youth | Interdisciplinary | Outpatient |
| Simons, Laura E., Lauren E. Harrison, Derek B. Boothroyd, Gomathy Parvathinathan, Amanda R. van Orden, Shannon F. O’Brien, Deborah Schofield, Joshua Kraindler, Rupendra Shrestha, Johan W. S. Vlaeyen, und Rikard K. Wicksell. 2024. „A Randomized Controlled Trial of Graded Exposure Treatment (GET Living) for Adolescents with Chronic Pain“. *Pain* 165(1):177–91. doi:10.1097/j.pain.0000000000003010. | RCT | Mixed Chronic Pain | Chronic Primary Pain | Youth | Interdisciplinary | Outpatient |
| den Hollander, Marlies, Mariëlle Goossens, Jeroen de Jong, Joop Ruijgrok, Jan Oosterhof, Patrick Onghena, Rob Smeets, und Johan W. S. Vlaeyen. 2016. „Expose or Protect? A Randomized Controlled Trial of Exposure in Vivo vs Pain-Contingent Treatment as Usual in Patients with Complex Regional Pain Syndrome Type 1“. *Pain* 157(10):2318–29. doi:10.1097/j.pain.0000000000000651. | RCT | Complex Regional Pain Syndrome | Chronic Primary Pain | Adult | Interdisciplinary | Outpatient |
| Linton, Steven J., Katja Boersma, Markus Jansson, Thomas Overmeer, Karin Lindblom, und Johan W. S. Vlaeyen. 2008. „A Randomized Controlled Trial of Exposure in Vivo for Patients with Spinal Pain Reporting Fear of Work‐related Activities“. European Journal of Pain 12(6):722–30. doi:10.1016/j.ejpain.2007.11.001. | RCT | Spinal Pain | Chronic Primary Pain | Adult | Interdisciplinary | Outpatient |
| Ashar, Yoni K., Alan Gordon, Howard Schubiner, Christie Uipi, Karen Knight, Zachary Anderson, Judith Carlisle, Laurie Polisky, Stephan Geuter, Thomas F. Flood, Philip A. Kragel, Sona Dimidjian, Mark A. Lumley, und Tor D. Wager. 2022. „Effect of Pain Reprocessing Therapy vs Placebo and Usual Care for Patients with Chronic Back Pain: A Randomized Clinical Trial“. *JAMA Psychiatry* 79(1):13. doi:10.1001/jamapsychiatry.2021.2669. | RCT | Chronic Back Pain | Chronic Primary Pain | Adult | Interdisciplinary | Outpatient |
| Wicksell, R. K., Melin, L., Lekander, M., & Olsson, G. L. 2009. "Evaluating the effectiveness of exposure and acceptance strategies to improve functioning and quality of life in longstanding pediatric pain: A randomized controlled trial." *Pain*, 141, 248–257. https://doi.org/10.1016/j.pain.2008.11.006 | RCT | Mixed Chronic Pain | Chronic Primary Pain | Youth | Interdisciplinary | Outpatient |
| Kent, Peter, Terry Haines, Peter O’Sullivan, Anne Smith, Amity Campbell, Robert Schutze, Stephanie Attwell, J. P. Caneiro, Robert Laird, Kieran O’Sullivan, Alison McGregor, Jan Hartvigsen, Den-Ching A. Lee, Alistair Vickery, und Mark Hancock. 2023. „Cognitive Functional Therapy with or without Movement Sensor Biofeedback versus Usual Care for Chronic, Disabling Low Back Pain (RESTORE): A Randomised, Controlled, Three-Arm, Parallel Group, Phase 3, Clinical Trial“. *The Lancet* 401(10391):1866–77. doi:10.1016/S0140-6736(23)00441-5. | RCT | Chronic Back Pain | Chronic Primary Pain | Adult | Physiotherapy | Outpatient |
| Vibe Fersum K, O’Sullivan P, Skouen JS, Smith A, Kvåle A. 2013. "Efficacy of classification-based cognitive functional therapy in patients with non-specific chronic low back pain: a randomized controlled trial." *Eur J Pain* 17: 916–28. doi: 10.1002/j.1532-2149.2012.00252.x | RCT | Chronic Back Pain | Chronic Primary Pain | Adult | Physiotherapy | Outpatient |
| O’Keeffe M, O’Sullivan P, Purtill H, Bargary N, O’Sullivan K. 2020. "Cognitive functional therapy compared with a group-based exercise and education intervention for chronic low back pain: a multicentre randomised controlled trial (RCT)." *Br J Sports Med* 54: 782–89. doi: 10.1136/bjsports-2019-100780 | RCT | Chronic Back Pain | Chronic Primary Pain | Adult | Physiotherapy | Outpatient/Group |
| Castro J, Correia L, Donato BS, et al. 2020. "Cognitive functional therapy compared with core exercise and manual therapy in patients with chronic low back pain: randomised controlled trial." *Pain* 163: 2430–37. doi: 10.1097/j.pain.0000000000002644 | RCT | Chronic Back Pain | Chronic Primary Pain | Adult | Physiotherapy | Outpatient |
| Glombiewski, Julia Anna, Sebastian Holzapfel, Jenny Riecke, Johan W. S. Vlaeyen, Jeroen de Jong, Gunnar Lemmer, und Winfried Rief. 2018. „Exposure and CBT for Chronic Back Pain: An RCT on Differential Efficacy and Optimal Length of Treatment.“ *Journal of Consulting and Clinical Psychology* 86(6):533–45. doi:10.1037/ccp0000298. | RCT | Chronic Back Pain | Chronic Primary Pain | Adult | Psychology | Outpatient |
| Woods, Marc P., und Gordon J. G. Asmundson. 2008. „Evaluating the Efficacy of Graded in Vivo Exposure for the Treatment of Fear in Patients with Chronic Back Pain: A Randomized Controlled Clinical Trial“. *Pain* 136(3):271–80. doi:10.1016/j.pain.2007.06.037. | RCT | Chronic Back Pain | Chronic Primary Pain | Adult | Psychology | Outpatient |
| Hedman-Lagerlöf, Maria, Erik Hedman-Lagerlöf, Erland Axelsson, Brjánn Ljótsson, Johanna Engelbrektsson, Sofia Hultkrantz, Karolina Lundbäck, Daniel Björkander, Rikard K. Wicksell, Ida Flink, und Erik Andersson. 2018. „Internet-Delivered Exposure Therapy for Fibromyalgia: A Randomized Controlled Trial“. *The Clinical Journal of Pain* 34(6):532–42. doi:10.1097/AJP.0000000000000566. | RCT | Fibromyalgia | Chronic Primary Pain | Adult | Psychology | Digital |
| Hedman-Lagerlöf M, Gasslander N, Ahnlund Hoffmann A, Bragesjö M, Etzell A, Ezra S, Frostell E, Hedman-Lagerlöf E, Ivert C, Liliequist B, Ljótsson B, Hoppe JM, Palmgren J, Spansk E, Sundström F, Särnholm J, Tzavara G, Buhrman M, Axelsson E. 2023. "Effect of exposure-based vs traditional cognitive behavior therapy for fibromyalgia: a two-site single-blind randomized controlled trial." Pain 165(6):1278-1288. doi: 10.1097/j.pain.0000000000003128. | RCT | Fibromyalgia | Chronic Primary Pain | Adult | Psychology | Digital |
| Boersma, Katja, Martin Södermark, Hugo Hesser, Ida K. Flink, Björn Gerdle, und Steven J. Linton. 2019. „Efficacy of a Transdiagnostic Emotion-Focused Exposure Treatment for Chronic Pain Patients with Comorbid Anxiety and Depression: A Randomized Controlled Trial“. *Pain* 160(8):1708–18. doi:10.1097/j.pain.0000000000001575. | RCT | Chronic Pain Patients with Comorbid Anxiety and Depression | Chronic Primary Pain | Adult | Psychology | Outpatient |
| Simons, Laura E., Johan W. S. Vlaeyen, Lies Declercq, Allison M. Smith, Justin Beebe, Melinda Hogan, Eileen Li, Corey A. Kronman, Farah Mahmud, Jenelle R. Corey, Christine B. Sieberg, und Christine Ploski. 2020. „Avoid or Engage? Outcomes of Graded Exposure in Youth with Chronic Pain Using a Sequential Replicated Single-Case Randomized Design“. *Pain* 161(3):520–31. doi:10.1097/j.pain.0000000000001735. | SCED | Mixed Chronic Pain | Chronic Primary Pain | Youth | Interdisciplinary | Outpatient |
| de Jong, Jeroen R., Johan W. S. Vlaeyen, Patrick Onghena, Corine Cuypers, Marlies den Hollander, und Joop Ruijgrok. 2005. „Reduction of Pain-Related Fear in Complex Regional Pain Syndrome Type I: The Application of Graded Exposure in Vivo“. *Pain* 116(3):264–75. doi:10.1016/j.pain.2005.04.019. | SCED | Complex Regional Pain Syndrome | Chronic Primary Pain | Adult | Interdisciplinary | Outpatient |
| de Jong, Jeroen R., Karoline Vangronsveld, Madelon L. Peters, Mariëlle E. J. B. Goossens, Patrick Onghena, Isis Bulté, und Johan W. S. Vlaeyen. 2008. „Reduction of Pain-Related Fear and Disability in Post-Traumatic Neck Pain: A Replicated Single-Case Experimental Study of Exposure In Vivo“. *The Journal of Pain* 9(12):1123–34. doi:10.1016/j.jpain.2008.06.015. | SCED | Post-Traumatic Neck Pain | Chronic Primary Pain | Adult | Interdisciplinary | Outpatient |
| de Jong, Jeroen R., Johan W. S. Vlaeyen, Marjon van Eijsden, Christoph Loo, und Patrick Onghena. 2012. „Reduction of Pain-Related Fear and Increased Function and Participation in Work-Related Upper Extremity Pain (WRUEP): Effects of Exposure in Vivo“. *Pain* 153(10):2109–18. doi:10.1016/j.pain.2012.07.001. | SCED | Work-Related Upper Extremity Pain | Chronic Primary Pain | Adult | Interdisciplinary | Outpatient |
| Boersma K, Linton S, Overmeer T, Jansson M, Vlaeyen J, de Jong J. 2004. "Lowering fear-avoidance and enhancing function through exposure in vivo. A multiple baseline study across six patients with back pain." *Pain* 108(1-2):8-16. doi: 10.1016/j.pain.2003.03.001. | SCED | Chronic Back Pain | Chronic Primary Pain | Adult | Interdisciplinary | Outpatient |
| Vlaeyen JWS, de Jong J, Geilen M, Heuts PHTG, van Breukelen G. 2002. The treatment of fear of movement/(re)injury in chronic low back pain: further evidence on the effectiveness of exposure in vivo. *Clin J Pain* 18:251–61. doi: 10.1097/00002508-200207000-00006. | SCED | Chronic Back Pain | Chronic Primary Pain | Adult | Interdisciplinary | Outpatient |
| Linton SJ, Overmeer T, Janson M, Vlaeyen JWS, de Jong JR. 2002. "Graded invivo exposure treatment for fear-avoidant pain patients with function disability: a case study." *Cogn Behav The*r 31:49–58. doi: 10.1080/16506070252959481 | SCED | Chronic Back Pain | Chronic Primary Pain | Adult | Interdisciplinary | Outpatient |
| de Jong JR, Vlaeyen JW, Onghena P, Goossens ME, Geilen M, Mulder H. 2005. "Fear of movement/(re)injury in chronic low back pain: education or exposure in vivo as mediator to fear reduction?" *Clin J Pain* 21(1):9-17; discussion 69-72. doi: 10.1097/00002508-200501000-00002. | SCED | Chronic Back Pain | Chronic Primary Pain | Adult | Interdisciplinary | Outpatient |
| Caneiro, J.P., O’Sullivan, P. Tan, J.-S., Klem, N.R., de Oliveira, B.I.R., Choong, P.F., Dowsey, M., Bunzli, S., Smith, A. 2024. "Process of change for people with knee osteoarthritis undergoing cognitive functional therapy: A replicated single-case experimental design study." *Disability and Rehabilitation* 46:11, 2348-2364, doi: 10.1080/09638288.2023.2221459. | SCED | Knee Osteoarthritis | Chronic Primary Pain | Adult | Physiotherapy | Outpatient |
| Al Sharaa, Haya, Sara Laureen Bartels, Afra S. Taygar, Linnéa Engman, Suzanne Petersson, Ida Flink, Katja Boersma, Lance M. McCracken, Laura Simons, Johan W. S. Vlaeyen, Patrick Onghena, und Rikard K. Wicksell. 2025. „Individual‐level Effects of a Digital Behavioural Treatment for Chronic Pain: Proof‐of‐concept of a Single‐case Experimental Design Study“. European Journal of Pain 29(10):e70128. doi:10.1002/ejp.70128. | SCED | Mixed Chronic Pain | Chronic Primary Pain | Adult | Psychology | Digital |
| Schemer L, Vlaeyen JWS, Doerr JM, Skoluda N, Nater UM, Rief W, Glombiewski JA. 2018. "Treatment processes during exposure and cognitive-behavioral therapy for chronic back pain: A single-case experimental design with multiple baselines." *Behav Res Ther* 108:58-67. doi: 10.1016/j.brat.2018.07.002. | SCED | Chronic Back Pain | Chronic Primary Pain | Adult | Psychology | Outpatient |
| Linton SJ, Fruzzetti AE. 2014. "A hybrid emotion-focused exposure treatment for chronic pain: A feasibility study." *Scand J Pain* 5(3):151-158. doi: 10.1016/j.sjpain.2014.05.008. | SCED | Chronic Back Pain | Chronic Primary Pain | Adult | Psychology | Outpatient |
| Hechler, Tanja, Michael Dobe, Uta Damschen, Markus Blankenburg, Sandra Schroeder, Joachim Kosfelder, und Boris Zernikow. 2010. „The Pain Provocation Technique for Adolescents with Chronic Pain: Preliminary Evidence for Its Effectiveness“. *Pain Medicine* 11(6):897–910. doi:10.1111/j.1526-4637.2010.00839.x. | Uncontrolled Trial | Mixed Chronic Pain | Chronic Primary Pain | Youth | Interdisciplinary | Inpatient |
| Wicksell, R. K., Melin, L., & Olsson, G. L. 2007. "Exposure and acceptance in the rehabilitation of adolescents with idiopathic chronic pain: A pilot study." *European Journal of Pain* 11, 267–274. https://doi.org/10.1016/j.ejpain.2006. 02.012. | Uncontrolled Trial | Mixed Chronic Pain | Chronic Primary Pain | Youth | Interdisciplinary | Outpatient |
| Fishbein, Joel N., Nathaniel M. Schuster, Alpha Anders, Ariel M. Portera, und Matthew S. Herbert. 2025. „Pain Reprocessing Therapy for Migraine: A Case Series“. *Headache: The Journal of Head and Face Pain* 65(9):1660–65. doi:10.1111/head.15043. | Uncontrolled Trial | Migraine | Chronic Primary Pain | Adult | Medicine | Outpatient |
| Hennessy, Rebecca White, Deanna Rumble, Mike Christian, David A. Brown, und Zina Trost. 2020. „A Graded Exposure, Locomotion-Enabled Virtual Reality App during Walking and Reaching for Individuals with Chronic Low Back Pain: Cohort Gaming Design“. *JMIR Serious Games* 8(3):e17799. doi:10.2196/17799. | Uncontrolled Trial | Chronic Back Pain | Chronic Primary Pain | Adult | N/A | Virtual Reality |
| Sturgeon, John, Zina Trost, Yoni K. Ashar, Mark A. Lumley, Howard Schubiner, Daniel Clauw, und Afton L. Hassett. 2025. „Brief Pain Reprocessing Therapy for Fibromyalgia: A Feasibility, Acceptability, and Preliminary Efficacy Pilot“. *Regional Anesthesia & Pain Medicine* rapm-2025-107076. doi:10.1136/rapm-2025-107076. | Uncontrolled Trial | Fibromyalgia | Chronic Primary Pain | Adult | Psychology | Outpatient |
| Zetterqvist, Vendela, Charlotte Gentili, Jenny Rickardsson, Isabel Sörensen, und Rikard K. Wicksell. 2020. „Internet-Delivered Acceptance and Commitment Therapy for Adolescents with Chronic Pain and Their Parents: A Nonrandomized Pilot Trial.“ *Journal of Pediatric Psychology* 45(9):990–1004. doi:10.1093/jpepsy/jsaa060. | Uncontrolled Trial | Mixed Chronic Pain | Chronic Primary Pain | Youth | Psychology | Digital |
| Ter Kuile, Moniek M., Reinhilde Melles, H. Ellen de Groot, Charlotte C. Tuijnman-Raasveld, und Jacques J. D. M. van Lankveld. 2013. „Therapist-Aided Exposure for Women with Lifelong Vaginismus: A Randomized Waiting-List Control Trial of Efficacy.“ *Journal of Consulting and Clinical Psychology* 81(6):1127–36. doi:10.1037/a0034292. | RCT | Vaginismus | Female Genitopelvic Pain | Adult | Psychology | Outpatient |
| Engman, Linnéa, Moniek M. Ter Kuile, Steven J. Linton, Elin Ekholm, Charlotte C. Tuijnman-Raasveld, und Ida K. Flink. 2022. „An Initial Proof of Concept: A Replicated Single-Case Study of a CBT Group Treatment with Partner Involvement for Vulvodynia“. *Cognitive Behaviour Therapy* 51(6):503–19. doi:10.1080/16506073.2022.2086907. | SCED | Vulvodynia | Female Genitopelvic Pain | Adult | Psychology | Outpatient/Group |
| Lalouni, Maria, Brjánn Ljótsson, Marianne Bonnert, Richard Ssegonja, Marc Benninga, Johan Bjureberg, Jens Högström, Hanna Sahlin, Magnus Simrén, Inna Feldman, Erik Hedman-Lagerlöf, Eva Serlachius, und Ola Olén. 2019. „Clinical and Cost Effectiveness of Online Cognitive Behavioral Therapy in Children with Functional Abdominal Pain Disorders“. *Clinical Gastroenterology and Hepatology* 17(11):2236-2244.e11. doi:10.1016/j.cgh.2018.11.043. | RCT | Functional Abdominal Pain | Gastrointestinal symptoms | Youth | Psychology | Digital |
| Kikuchi, Shino, Yuki Oe, Yuri Ito, Takashi Sozu, Yohei Sasaki, Masatsugu Sakata, Yan Luo, Ethan Sahker, Masaru Horikoshi, Hiroshi Seno, und Toshi A. Furukawa. 2022. „Group Cognitive-Behavioral Therapy with Interoceptive Exposure for Drug-Refractory Irritable Bowel Syndrome: A Randomized Controlled Trial“. *American Journal of Gastroenterology* 117(4):668–77. doi:10.14309/ajg.0000000000001664. | RCT | Irritable Bowel Syndrome | Gastrointestinal symptoms | Adult | Interdisciplinary | Outpatient/Group |
| Bonnert, Marianne, Ola Olén, Maria Lalouni, Marc A. Benninga, Matteo Bottai, Johanna Engelbrektsson, Erik Hedman, Fabian Lenhard, Bo Melin, Magnus Simrén, Sarah Vigerland, Eva Serlachius, und Brjánn Ljótsson. 2017. „Internet-Delivered Cognitive Behavior Therapy for Adolescents with Irritable Bowel Syndrome: A Randomized Controlled Trial“. *American Journal of Gastroenterology* 112(1):152–62. doi:10.1038/ajg.2016.503. | RCT | Irritable Bowel Syndrome | Gastrointestinal symptoms | Adult | Psychology | Digital |
| Craske, Michelle G., Kate B. Wolitzky-Taylor, Jennifer Labus, Stephen Wu, Michael Frese, Emeran A. Mayer, und Bruce D. Naliboff. 2011. „A Cognitive-Behavioral Treatment for Irritable Bowel Syndrome Using Interoceptive Exposure to Visceral Sensations“. *Behaviour Research and Therapy* 49(6–7):413–21. doi:10.1016/j.brat.2011.04.001. | RCT | Irritable Bowel Syndrome | Gastrointestinal symptoms | Adult | Psychology | Outpatient |
| Ljótsson, Brjánn, Gerhard Andersson, Erik Andersson, Erik Hedman, Perjohan Lindfors, Sergej Andréewitch, Christian Rück, und Nils Lindefors. 2011. „Acceptability, Effectiveness, and Cost-Effectiveness of Internet-Based Exposure Treatment for Irritable Bowel Syndrome in a Clinical Sample: A Randomized Controlled Trial“. *BMC Gastroenterology* 11(1):110. doi:10.1186/1471-230X-11-110. | RCT | Irritable Bowel Syndrome | Gastrointestinal symptoms | Adult | Psychology | Digital |
| Ljótsson, Brjánn, Lisa Falk, Amanda Wibron Vesterlund, Erik Hedman, Perjohan Lindfors, Christian Rück, Timo Hursti, Sergej Andréewitch, Liselotte Jansson, Nils Lindefors, und Gerhard Andersson. 2010. „Internet-Delivered Exposure and Mindfulness Based Therapy for Irritable Bowel Syndrome – A Randomized Controlled Trial“. *Behaviour Research and Therapy* 48(6):531–39. doi:10.1016/j.brat.2010.03.003. | RCT | Irritable Bowel Syndrome | Gastrointestinal symptoms | Adult | Psychology | Digital |
| Ljótsson, Brjánn, Erik Hedman, Erik Andersson, Hugo Hesser, Perjohan Lindfors, Timo Hursti, Sara Rydh, Christian Rück, Nils Lindefors, und Gerhard Andersson. 2011. „Internet-Delivered Exposure-Based Treatment vs. Stress Management for Irritable Bowel Syndrome: A Randomized Trial“. *American Journal of Gastroenterology* 106(8):1481–91. doi:10.1038/ajg.2011.139. | RCT | Irritable Bowel Syndrome | Gastrointestinal symptoms | Adult | Psychology | Digital |
| Ljótsson, Brjánn, Hugo Hesser, Erik Andersson, Jeffrey M. Lackner, Samir El Alaoui, Lisa Falk, Kristina Aspvall, Josefin Fransson, Klara Hammarlund, Anna Löfström, Sanna Nowinski, Perjohan Lindfors, und Erik Hedman. 2014. „Provoking Symptoms to Relieve Symptoms: A Randomized Controlled Dismantling Study of Exposure Therapy in Irritable Bowel Syndrome“. *Behaviour Research and Therapy* 55:27–39. doi:10.1016/j.brat.2014.01.007. | RCT | Irritable Bowel Syndrome | Gastrointestinal symptoms | Adult | Psychology | Digital |
| Wallén, Hugo, Brjánn Ljótsson, Perjohan Lindfors, Erik Forsell, Hugo Hesser, und Cecilia Svanborg. 2025. „Internet-Delivered Exposure-Based Cognitive Behavior Therapy for Irritable Bowel Syndrome: A Clinical Effectiveness Study“. *American Journal of Gastroenterology* 120(4):856–63. doi:10.14309/ajg.0000000000003059. | Uncontrolled Trial | Irritable Bowel Syndrome | Gastrointestinal symptoms | Adult | Psychology | Digital |
| Wallén, Hugo, Brjánn Ljótsson, Cecilia Svanborg, Sara Rydh, Lisa Falk, und Perjohan Lindfors. 2022. „Exposure Based Cognitive Behavioral Group Therapy for IBS at a Gastroenterological Clinic – a Clinical Effectiveness Study“. *Scandinavian Journal of Gastroenterology* 57(8):904–11. doi:10.1080/00365521.2022.2047220. | Uncontrolled Trial | Irritable Bowel Syndrome | Gastrointestinal symptoms | Adult | Psychology | Outpatient/Group |
| Hecker, Lynn, Skye King, Sven Stapert, Chantal Geusgens, Marlies den Hollander, Britt Fleischeuer, und Caroline van Heugten. 2025. „Can Exposure Therapy Be Effective for Persistent Post-Concussion Symptoms? A Nonconcurrent Multiple Baseline Design across 4 Cases“. *Journal of Head Trauma Rehabilitation*. doi:10.1097/HTR.0000000000001023. | SCED | Persistent Post-Concussion Symptom | Persistent Post-Concussion Symptoms | Adult | Psychology | Outpatient |
| King, Skye, Sven Z. Stapert, Ieke Winkens, Joukje van der Naalt, Caroline M. van Heugten, und Marleen M. Rijkeboer. 2024. „Efficacy of an Intensive Exposure Intervention for Individuals with Persistent Concussion Symptoms Following Concussion: A Concurrent Multiple Baseline Single-Case Experimental Design (SCED) Study“. *Journal of Head Trauma Rehabilitation* 39(5):E419–29. doi:10.1097/HTR.0000000000000942. | SCED | Persistent Post-Concussion Symptom | Persistent Post-Concussion Symptoms | Adult | Psychology | Outpatient |
| King, Skye, Ieke Winkens, Joukje van der Naalt, Caroline M. van Heugten, und Marleen M. Rijkeboer. 2025. „Intensive Exposure Therapy for Individuals with Persistent Concussion Symptoms Following Concussion: A Replicated Single-Case Experimental Design (SCED) Study“. *Brain Injury* 1–9. doi:10.1080/02699052.2025.2544624. | SCED | Persistent Post-Concussion Symptom | Persistent Post-Concussion Symptoms | Adult | Psychology | Outpatient |
| Cima, Rilana Ff, Iris H. Maes, Manuela A. Joore, Dyon Jwm Scheyen, Amr El Refaie, David M. Baguley, Lucien Jc Anteunis, Gerard Jp van Breukelen, und Johan Ws Vlaeyen. 2012. „Specialised Treatment Based on Cognitive Behaviour Therapy versus Usual Care for Tinnitus: A Randomised Controlled Trial“. *The Lancet* 379(9830):1951–59. doi:10.1016/S0140-6736(12)60469-3. | RCT | Tinnitus | Tinnitus | Adult | Interdisciplinary | Outpatient |
| Fuller, Thomas E., Gerard J. P. van Breukelen, Johan W. S. Vlaeyen, und Rilana F. F. Cima. 2022. „Pragmatic Uncontrolled Study of Specialized Cognitive Behavioral Therapy for Adults with Chronic Tinnitus“. *Ear & Hearing* 43(6):1893–1903. doi:10.1097/AUD.0000000000001226. | Uncontrolled Trial | Tinnitus | Tinnitus | Adult | Interdisciplinary | Outpatient |
